# Synthesis of double-clickable functionalised graphene oxide for biological applications†
†Electronic supplementary information (ESI) available. See DOI: 10.1039/c5cc05412e



**DOI:** 10.1039/c5cc05412e

**Published:** 2015-08-21

**Authors:** Kuo-Ching Mei, Noelia Rubio, Pedro M. Costa, Houmam Kafa, Vincenzo Abbate, Frederic Festy, Sukhvinder S. Bansal, Robert C. Hider, Khuloud T. Al-Jamal

**Affiliations:** a Institute of Pharmaceutical Science , King's College London , Franklin-Wilkins Building , 150 Stamford Street , London SE1 9NH , UK . Email: khuloud.al-jamal@kcl.ac.uk; b Biomaterials and Biomimetics Department , King's College London Dental Institute , London SE1 9RT , UK

## Abstract

Azide- and alkyne-double functionalised graphene oxide (Click^2^ GO) was synthesised and characterised with attenuated total reflectance Fourier transform infrared spectroscopy (ATR-FTIR), thermogravimetric analysis (TGA) and Raman spectroscopy. Fourteen-percentage increase in azide content was found, after pre-treatment of GO with *meta*-chloroperoxybenzoic acid (mCPBA), determined with elemental analysis. No effect on A549 cell viability was found, up to 100 μg mL^–1^ and 72 h of incubation, determined with the modified lactate dehydrogenase (mLDH) assay. Two sequential copper(i) catalysed azide–alkyne cycloaddition (CuAAC) reactions were performed to conjugate the propargyl-modified blood–brain barrier targeting peptide Angiopep-2, and a bis-azide polyethylene glycol (*M*_W_ = 3500), to the Click^2^ GO. The final conjugate was characterised with ATR-FTIR and TGA.

## Abstract 2

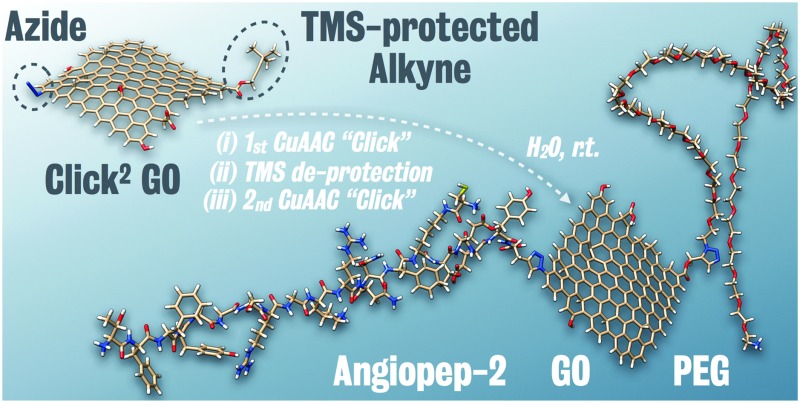
Azide- and alkyne-double functionalised graphene oxide (Click^2^ GO) was synthesised and characterised with ATR-FTIR, TGA, and Raman spectroscopy.

Graphene and graphene oxide (GO) are single atom thick, 2-D carbon structures that have recently been widely used in many fields including drug delivery,^1,2^ gas separation (as membrane material),^3^ electrochemical sensing, biosensing,^4,5^ bio-imaging and photothermal therapy.^6^ One of the very popular methods to functionalise nanomaterials for drug delivery is *via* click chemistry, in particular, the copper(i) catalysed azide–alkyne cycloaddition (CuAAC) and strained-promoted azide–alkyne cycloaddition (SPAAC).^7^ CuAAC click chemistry has also been used to functionalise carbon based nano-materials *e.g.* fullerene,^8^ carbon nanotubes,^9^ and graphene.^10^ Alkyne-modified GO (GO-alkyne) has been used to prepare functionalised GO for different applications *e.g.* for CO_2_ absorption, as photo-active sheets, and as efficient absorbents for dye removal.^10–17^ The introduced alkyne, however, also increased GO's hydrophobicity, making it less dispersible in aqueous media. Only one study reported alkyne-modified GO for drug delivery application after conjugation with water-dispersible polymers, used to restore water dispersibility.^18^ Double functionalised GO contains both azide and protected alkyne has not been reported. Herein, we propose to synthesise GO containing double clicking sites *i.e.* azide and TMS-protected alkyne groups, refereed to here as double-clickable or Click^2^ GO, for biological applications. This approach allows sequential CuAAC reactions, on GO, in aqueous media. Sequential CuAAC has been demonstrated on fullerene,^8^ but not on graphene based material. Compared to fullerene, GO provides a significantly larger surface area and superior water dispersibility thus offering more potential for biomedical applications. As shown in Scheme 1, simple quick GO functional groups interconversion followed by two sequential CuAAC clicks for double-GO-functionalisation is presented.

**Scheme 1:**
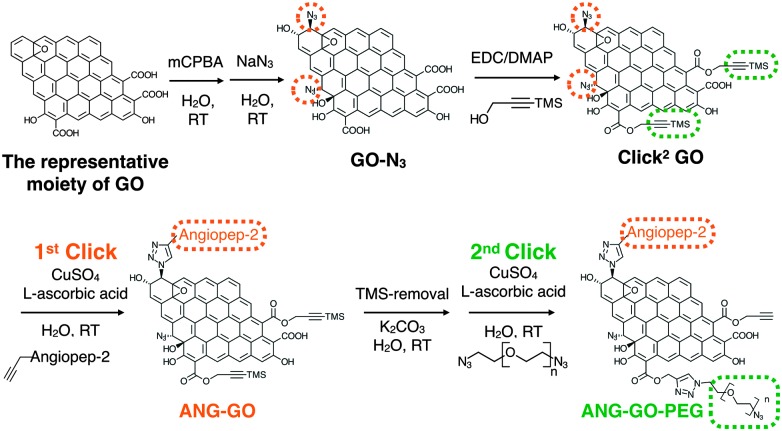
Sequential functionalisation of GO using two CuAAC click reactions.

In previous reports, GO-N_3_ was generated by introducing azide groups onto the carboxylic groups of GO through amidation with 3-azidopropan-1-amine (NH_2_(CH_2_)_3_N_3_)^19^ or *via* nucleophilic substitution using 2-chloroethyl isocyanate (Cl(CH_2_)_2_NCO).^20^ Eigler *et al.* reported azide functionalisation of GO using NaN_3_.^21^ The reaction was achieved by the substitution of organosulfate and ring opening of epoxy groups on GO with azide when reacted with NaN_3_. GO used in the same study was prepared using the modified Hummer's method. Azide functionlisation of epoxide group can occur *via* 1,2-epoxide ring opening using NaN_3_ generating 1,2-azidoalcohols.^22,23^ In the current study, azide introduction was achieved using epoxide groups on GO leaving the carboxylic groups available for subsequent functionalisation, *i.e.* introduction of alkyne groups. Using this approach, it is also expected that the additional hydroxyl groups generated from azidolysis can improve the water dispersibility of GO-N_3_. Azide content was enriched by further epoxidising GO using *meta*-chloroperoxybenzoic acid (mCPBA), which has been reported to epoxidise fullerenes^24^ and CNTs^25–27^ but not GO. After azide groups were introduced on GO, alkyne groups were introduced *via* Steglich esterification of the preserved carboxylic groups^28^ with trimethylsilyl (TMS)-protected propargyl alcohol, yielding double functionalised GO or Click^2^ GO.

One of the commonly used methods to prepare GO is the chemical exfoliation of graphite by “Hummer's method” firstly reported by Hummers and Offeman, in 1958.^29^ Many modifications have been proposed since then. This method involves several oxidation and hydration steps carried out at different temperatures. An improved Hummer's method was published by Kovtyukhova *et al.* ^30^ In their method, graphite powder was pre-oxidised before proceeding with the modified Hummer's method. In addition to that, the addition of NaNO_3_ as an oxidising agent, was omitted. A modified Kovtyukhova–Hummer's method was used to synthesis GO in our recent work and in this study.^31^ Steps carried out in our study are described in ESI† Methods (Fig. S1) and schematically summarised in Scheme S1. In brief, Kovtyukhova–Hummer's method was adapted with further modification including: (i) NaNO_3_ was kept in this reaction, (ii) the water was added in portions at controlled temperature, and (iii) a heating step was introduced at H_2_O_2_ addition step prior to the washing and purification step. Major steps involved pre-oxidation of graphite, low-, medium-, high-temperature treatment stages followed by washing and purification. At the end, a water-dispersible dark-brown GO suspension was obtained. The final concentration of GO in the dispersion was 6.84 mg mL^–1^, as determined by thermogravimetric analysis (TGA) (eqn (S1), ESI†). The stock GO dispersion was then diluted to the appropriate concentration when desired. The modified Hummer's reported by Ali-Boucetta was used as a control method,^32^ yielding ∼2 mg GO from 300 mg graphite. In contrast, modified Kovtyukhova–Hummer's method generated ∼5470 mg GO from 4000 mg graphite (Table S1, ESI†). Elemental analysis (C, H, N, O) was performed to determine the oxidation status of the pre-oxidised graphite and GO (Table S2, ESI†). Based on final carbon content, % GO yield was calculated to be ∼55.63% (eqn (S2) and (S3), ESI†).

The as synthesized GO was fully characterised using thermogravimetric analysis (TGA), Raman spectroscopy,^33–35^ TEM and AFM. In brief, the final residual weight at 978 °C was 99.36%, 94.15% and 38.16% for graphite, pre-oxidised graphite and GO, respectively. Raman spectroscopy confirmed the generation of GO with G peak at 1598 cm^–1^, D peak at 1326 cm^–1^. The respective *I*_D_/*I*_G_ (peak height) values of graphite and GO were 0.21 ± 0.01 and 1.22 ± 0.01, respectively (*n* = 3). Detailed information can be found in ESI† (SI Method and Fig. S2–S4).

GO-N_3_ was then prepared using NaN_3_ and 1,2-epoxide ring opening reaction as described in ESI,† Methods (Scheme S2). GO-N_3_ was characterised by Raman spectroscopy and attenuated total reflectance Fourier transform infrared (ATR-FTIR) using the methods described in SI. A unique azide peak at 2121 cm^–1^ was found when azide groups were introduced on GO. Free azide peak was detected at 2053 cm^–1^ when the purification was not properly performed (Fig. S5, ESI†). All the oxygen functionalities found in GO were also observed in GO-N_3_ except that the intensity of hydroxyl related peaks was higher (3200, 1227, and 1036 cm^–1^) than that in GO (Fig. S6A, ESI†). The presence of a single azide peak at 2121 cm^–1^ confirmed the elimination of free azides. Raman spectra were normalised to the G peak (intensity equals 1) (Fig. S6B, ESI†). Higher *I*_D_/*I*_G_ (peak height) ratio was obtained for GO-N_3_ (1.30 ± 0.05, *n* = 3) than GO (1.25 ± 0.01, *n* = 3). The difference, however, was not significant with *t*(2) = –1.62, *p*-value = 0.246. The presence of azide group was further confirmed by Staudinger-Ninhydrin assay (ESI,† Methods, Scheme S3, Fig. S6C).^36,37^ In order to increase the azide group content on GO-N_3_, *m*-chloroperoxybenzoic acid (mCPBA), a strong oxidising reagent, commonly used to epoxidise alkenes was used to introduce more epoxide groups on GO. Epoxidation of GO is generally not required as epoxides are known to be naturally present when graphite is oxidised to GO.^38,39^ Direct epoxidation of GO with H_2_O_2_, however, has been reported to generate ultra-small graphene quantum dots (GQD) *via* epoxide ring opening.^40–42^ To the best of our knowledge, direct epoxidation of GO with mCPBA has not been reported in the literature. In this work, mCPBA was therefore used to enrich epoxide content on GO surface to form epo-GO. Methods of epo-GO preparation is summarised in ESI,† Methods and presented in Scheme S4. Elemental analysis (C, H, N, O) was performed to determine N% in GO and GO-N_3_ derivatives (Table S3, ESI†). The N% for GO, GO-N_3_ (–mCPBA), and GO-N_3_ (+mCPBA) were <0.10, 0.71, and 0.81%, respectively. The mCPBA pre-treatment increased the azide content by 14%. The IR spectra of GO and GO-N_3_ (±mCPBA) are shown in Fig. 1A. The azide peak at 2120 and 2122 cm^–1^ was found for GO-N_3_ (–mCPBA) and GO-N_3_ (+mCPBA), respectively.

**Fig. 1:**
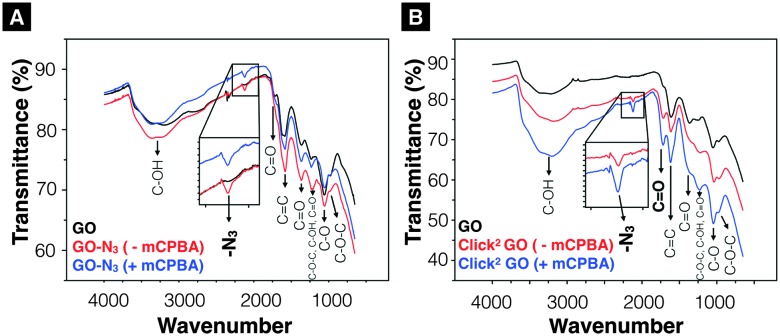
Infrared-transmittance spectra of GO-N_3_ derivatives and Click^2^ GO derivatives. (A) GO-N_3_ derivatives. The introduction of azide groups was confirmed at 2120 cm^–1^ (GO-N_3_ –mPCBA), 2122 cm^–1^ (GO-N_3_ +mCPBA). (B) Click^2^ GO derivatives. (Click^2^ GO +mCPBA). Enhanced C–OH peaks (3200 cm^–1^, 1227 cm^–1^ and 1036 cm^–1^) were observed. Full description of the peak locations can be found in ESI.†

After confirming the introduction of azide groups on GO, Click^2^ GO was prepared *via* Steglich esterification as described in ESI,† Methods and Scheme 1. Samples were prepared with or without mCPBA, to mimic GO-N_3_ preparation condition. The IR spectra of Click^2^ GO are shown in Fig. 1B. The azide peak at 2117 or 2120 cm^–1^ was found in Click^2^ GO (–mCPBA) or Click^2^ GO (+mCPBA), respectively. Similar to GO-N_3_, the epoxidation treatment slightly increased the wavenumber of azide peak. The alkyne signal of Click^2^ GO could not be picked up by Raman spectroscopy (Fig. S7, ESI†). This was expected as the –C≡C– stretching vibration on macromolecules generally has a weak IR transmittance. Furthermore, the peak location overlaps with the azide groups. The Raman spectra *I*_D_/*I*_G_ (peak height) ratio was 1.32 ± 0.03 and 1.28 ± 0.01 (*n* = 3) for Click^2^ GO, without and with mCPBA, respectively. TGA was used to characterise GO, GO-N_3_, and Click^2^ GO. As shown in Fig. 2, GO exhibited 4 stages of thermal-decomposition, represented by the 4 disconnected linear weight-loss intervals with curvy turning points. GO-N_3_ also showed 4 linear weight-loss intervals with relatively sharp turning points. A higher and a sharper weight-loss slope was found in the linear range Ib than in Ia. These changes could be due to the decomposition of azide groups when the temperature approaches 200 °C, as previously reported in the literature.^43^ Click^2^ GO also showed a deep and steep drop in weight within the linear range Ic, however; only two linear ranges were found in Click^2^ GO; the stage 4 decomposition observed in GO (IVa) and GO-N_3_ (IVb) could not be seen in Click^2^ GO. The introduction of TMS-protected propargyl alcohol on the carboxylic groups seems to have stabilised the GO at 800 °C or higher. Detailed information on slopes analysis is summarised in Tables S4–S6 (ESI†).

**Fig. 2:**
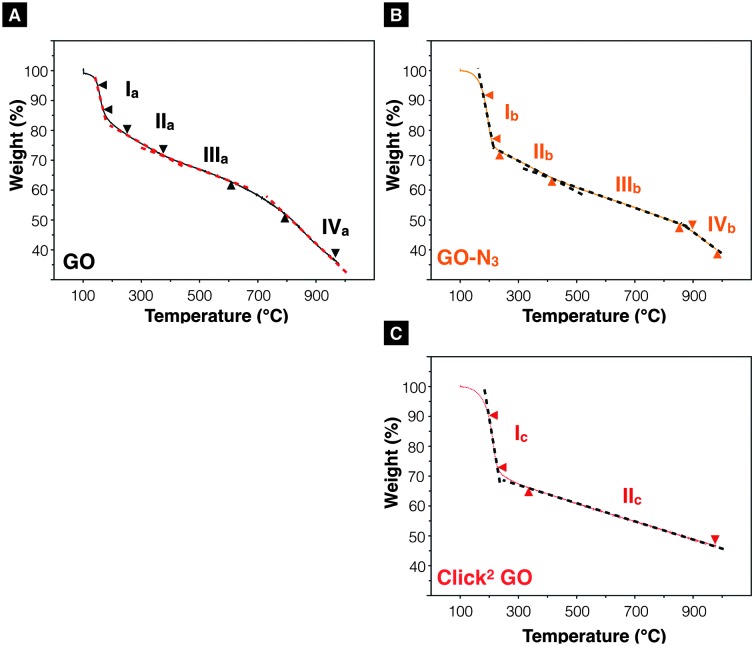
Thermogravimetric analysis. (A) GO and (B) GO-N_3_ exhibit four linear decomposition ranges when heated from 100–978 °C. A steeper curve was found in linear range Ib than Ia. (C) Click^2^ GO. Only two linear thermal-decomposition ranges were found throughout the heating range. It was noticed that the introduction of alkyne groups eliminated stage IV decomposition event observed in IVa and IVb.

In preparation of introducing the synthesised GO derivatives as potential drug carriers, a size reduction step was introduced by bath sonication up to 4–6 h. Transmission electron microscopy (TEM) and Atomic Force Microscopy (AFM) were used to analyse the flake surface area (Fig. S3 and S4, ESI†). Maximum/medium size reduction from 13.19 μm^2^/186 nm^2^ (prior sonication, *n* = 370) to 2.15 μm^2^/62 nm^2^ (after 4 h bath sonication, *n* = 621) was confirmed by AFM.

The Propargyl-modified blood–brain barrier targeting peptide angiopep-2 (TFFYGGSRGKRNNFKTEEYG)^44^ and bis-azide polyethylene glycol (N_3_–PEG_3500_–N_3_) were synthesised using Fmoc solid phase peptide synthesis and reaction with imidazole-1-sulfonyl azide hydrochloride,^45^ respectively (SI method, Fig. S8 and S9, ESI†). The as-synthesised Click^2^ GO was double-functionalised using two sequential CuAAC clicks. In brief, propargyl- modified angiopep-2 was firstly clicked to the azide groups of the GO yielding ANG-GO. The TMS-alkyne was then de-protected and clicked to N_3_–PEG_3500_–N_3_ yielding ANG-GO-PEG. Control click reactions were run in parallel. The first and second control click reactions were run lacking angiopep-2 and CuSO_4_/l-ascorbic acid, respectively. The latter was performed to evaluate the physical absorption of PEG on the GO. Conjugation of angiopep-2 was confirmed by the reduction in the azide IR intensity (2120 cm^–1^) and the appearance of amide I (1647 cm^–1^, C=O stretching vibration) and amide II (1515 cm^–1^, N–H bending vibration) signals on IR spectra of ANG-GO (Fig. 3A). The conjugation of N_3_–PEG_3500_–N_3_ to GO in ANG-GO-PEG was confirmed by the appearance of the two C–H stretching vibration signals at 2869 and 2920 cm^–1^ (Fig. 3A). TGA showed evidence of thermal decomposition between 200–300 °C, corresponding to angiopep-2, in both ANG-GO and ANG-GO-PEG. PEG decomposition on the other hand was observed at a temperature above 480 °C (Fig. 3B). The IR and TGA results of the control experiments are shown in Fig. S10 (ESI†) further confirming that sequential click reactions took place. The TEM images of ANG-GO and ANG-GO-PEG are shown in Fig. S11 and S12 (ESI†), respectively.

**Fig. 3:**
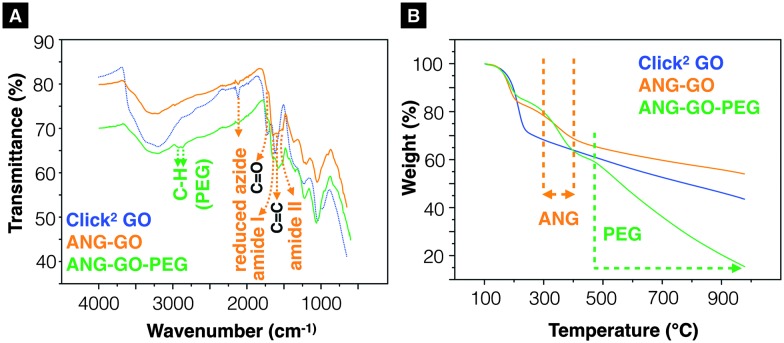
Characterisation of ANG-GO and ANG-GO-PEG. (A) Infrared-transmittance spectra and (B) thermogravimetric analysis of aniopep-2 functionalised Click^2^ GO (ANG-GO) and angiopep-2/PEG double-functionalised Click^2^ GO (ANG-GO-PEG).

Optical microscopy images of A549 incubated with GO, GO-N_3_, Click^2^ GO, ANG-GO, and ANG-GO-PEG were taken at 24 and 72 h time points. Cells incubated with functionalised GO showed normal morphology at all concentrations tested, while cells treated with DMSO appeared unhealthy and detached from the plate (Fig. S13, ESI†). Click^2^ GO then GO-N_3_ appeared to interact better with cells compared to plain GO sheets, as shown by the dark signals localised to cells. The extent of GO uptake was concentration and time-dependent (Fig. S14 and S15, ESI†). This interaction was further enhanced in both ANG-GO and ANG-GO-PEG (Fig. S16 and 17, ESI†). The modified lactate dehydrogenase (LDH) assay was used to assess the cytotoxicity of all GO derivatives on A549 cells (adenocarcinoma human alveolar basal epithelial cells) using the method described in SI.^46^ Cells were incubated with GO, GO-N_3_, Click^2^ GO, ANG-GO, and ANG-GO-PEG at 10, 50, and 100 μg mL^–1^ for 24 and 72 h. Dimethyl sulfoxide (DMSO) was used as a positive control. The cytotoxicity result from the mLDH assay is shown in Fig. 4. No significant effect on cell viability was observed in GO, CO-N_3_, and Click^2^ GO. Lower viability was observed in ANG-GO at 50 and 100 μg mL^–1^ at 72 h (*p* < 0.001). The viability of GO-ANG was restored when PEGylated (ANG-GO-PEG).

**Fig. 4:**
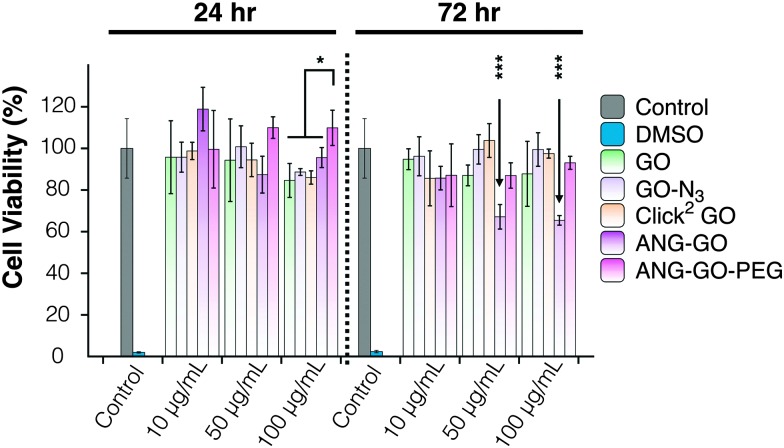
Modified LDH assay of A549 cells incubated with GO, GO-N_3_, Click^2^ GO, ANG-GO, and ANG-GO-PEG. The toxicity was assessed using the modified LDH assay. No significant effect on cell viability was observed in GO, CO-N_3_, and Click^2^ GO. Lower viability was observed in ANG-GO at 50 and 100 μg mL^–1^ at 72 h (*p* < 0.001). (*p < 0.05; ***p < 0.001). The viability was recovered in ANG-GO-PEG.

The double-clickable GO is reported for the first time, offering an eco-friendly method to perform CuAAC at room temperature in aqueous media, *i.e.*, organic solvent-free. This is particularly important for GO functionalization as the GO sheets do not disperse well in organic solvents with layers tend to fold-up. Water-based reaction is expected to offer maximum exposure of GO surface to the reactants.

In this study, GO was synthesised using a modified Kovtyukhova–Hummer's method.^31^ This method greatly improved the yield of GO (55.63%) when compared to the traditional modified Hummer's method (<1%). The azide-functionalisation of GO was achieved using NaN_3_. The azide content was boosted by 14% upon pre-treatment with mCPBA. Double functionalisation of Angiopep-2 and N_3_–PEG_3500_–N_3_ on Click^2^ GO using sequential CuAAC clicks was demonstrated. No major effect on cell viability was found for Click^2^ GO. Lower cell viability was found in ANG-GO at 50/100 μg mL^–1^ at 72 h, which was reversed in ANG-GO-PEG. Click^2^ GO is proposed here as an alternative graphene-based platform for applications in drug delivery.

K.-C.M. would like to thank King's College London for the Graduate School International Research Award (GSIRA) scholarship. Funding from Biotechnology and Biological Sciences Research Council (BB/J008656/1) and Wellcome Trust (WT103913MF) is acknowledged. H.K. was sponsored by the Atomic Energy Commission of Syria. P.M.C. is a Sir Henry Wellcome postdoctoral fellow. The author would also like to thank Dr K. L. Andrew Chan at King's College London for constructive discussions.
