# Supplementary material for: Synthesis of double-clickable functionalised graphene oxide for biological applications
Source: Chem Commun (Camb). 2015 Aug 21;51(81):14981–4. doi: 10.1039/c5cc05412e (PMC4594119; doi:10.1039/c5cc05412e)
Supplement: Supplementary file 1 [file CC-051-C5CC05412E-s001.pdf]

Electronic Supplementary Material (ESI) for Chemical Communications.
This journal is © The Royal Society of Chemistry 2015

Synthesis of double-clickable functionalised graphene oxide for biological applications

Kuo-Ching Mei,[a] Noelia Rubio,[a] Pedro M. Costa,[a] Houmam Kafa,[a] Vincenzo Abbate,[a] Frederic Festy,[b] Sukhvinder S. Bansal,[a] Robert C. Hider,[a] and Khuloud T. Al-Jamal[a*]

[a] *Institute of Pharmaceutical Science, King's College London, Franklin-Wilkins Building, 150 Stamford Street, London SE1 9NH, UK.*

[b] *Biomaterials and Biomimetics Department, King's College London Dental Institute, London SE1 9RT, UK.*

Keywords: graphene; click chemistry; nanomedicine; drug delivery; peptide

Email: khuloud.al-jamal@kcl.ac.uk

Supplementary Information

Material

Materials for Chemical Experiments

1-Ethyl-3-(3-dimethylaminopropyl)carbodiimide (EDC, cat: ANA29855) was obtained from Bioscience (UK). Graphite powder (cat: SP-1, batch: 04100, lot: 011705) was purchased from Bay Carbon, Inc. (USA). Silicone oil (cat: A122718, for oil baths, usable ranges from 40 °C to + 200 °C) was from Alfa Aesar®(UK). Filter paper (cat: 1001240) was from GE Whatman™ (UK). Ninhydrin (cat: sc203162) was from Santa Cruz Biotechnology (USA). H_2O_2 (cat: 202460010) and HNO_3 (cat: 1244660011) were purchased from Acros Organics (USA). Acetyl chloride (cat: 607-011-00-5) and sulfuryl chloride (SO_2Cl_2) (cat: 016016006) were purchased from Merck/VWR International (UK).

Deuterium oxide (cat: 1133660010), Fluoropore™ membrane, PTFE, hydrophobic, 0.22 µm (cat: FGLP04700), Isopore™ membrane, polycarbonate, hydrophilic, 0.22 µm (cat: GTTP04700) were ordered from Merck Millipore (UK). Acetone (cat: A/0600/17), dichloromethane (cat: D/1852/17), chloroform (cat: C/4960/17), and methanol (cat: M/4000/17) were purchased from Fisher Scientific (UK). HCl (cat: 258148), $NaNO_3$ (cat: 221341), $KMnO_4$ (cat: 60459), P_2O_5 (cat: 214701), NaN_3 (cat: S2002), PPh_3 (cat: 93092), KCN (cat: 60180), meta-chloroperoxybenzoic acid ≤ 77% (mCPBA, cat: 273031), 3-(trimethylsilyl)propargyl alcohol (TMS-protected propargyl alcohol, cat: 318558), 4-(Dimethylamino)pyridine (DMAP, cat: 522805), chloroform-d (cat: 151823), imidazole (cat: 12399) and phenol (cat: 242322) were all obtained from Sigma-Aldrich (UK). Polyoxyethylene-bis-amine (NH_2-$PEG_{3.5}$KDa-NH_2) was purchased from JENKEM Technology (USA).

Materials for Peptide Synthesis

Fmoc-Lys(Dde)-OH (cat#852057), Fmoc-Ser(tBu)-OH (cat#852019), FMOC-Asn(Trt)-OH (cat#852044), Fmoc-Ile-OH (cat#852010), Fmoc-Phe-OH (cat#852016), Fmoc-Glu(OtBu)-OH (cat#852009), Fmoc-Leu-OH (cat#852011), Fmoc-Thr(tBu)-OH (cat#852000), Fmoc-Tyr(tBu)-OH (cat#852020), Fmoc-

Arg(Pbf)-OH (cat#852067), Fmoc-Cys(Trt)-OH (cat#852008), Fmoc-Gly-OH (cat#852001), Fmoc-Lys(Boc)-OH (cat#852012), Fmoc-L-propargylglycine (cat#852360), Triisopropylsilane (cat#841359), NovaSyn®TGR resin (cat#855009) were purchased from Millipore (UK). N,N-Dimethylformamide (cat#D119-1) was purchased from ThermoFisher Scientific (UK). Phenol (cat#W322318), N,N-Dimethylformamide (cat#227056), 2,3,5-Collidine (cat#513261), O-(6-Chlorobenzotriazol-1-yl)-N,N,N′,N′-tetramethyluronium hexafluorophosphate (cat#04936), Piperidine (cat#104094), Thioanisole (cat#88470), and 2,2′(Ethylenedioxy)diethanethiol (cat#465178) were all obtained from Sigma-Aldrich (UK).

Materials for Biology Experiments

Fetal bovine serum (FBS) was obtained from First-Link, UK Ltd. Phosphate Buffered Saline (PBS) (10X, pH 7.4), Dulbecco's Modified Eagle Medium (DMEM), Minimum Essential Medium/HEPES, Penicillin-Streptomycin 100X, 0.05 % Trypsin-EDTA (1X) with Phenol Red, GlutaMAX™ Supplement were obtained from Invitrogen, Life Sciences (UK). CytoTox 96® Non-Radioactive Cytotoxicity Assay was obtained from Promega Corporation (UK).

Methods

Preparation of GO by modified Hummer's method

The modified Hummer's method reported by Ali-Boucetta *et al.*[1] was used initially to prepare GO for comparison only. In brief, graphite powder (300 mg) and sodium nitrate powder ($NaNO_3$, 150 mg) were dry-mixed in a 250 mL round bottom flask. Sulfuric acid (H_2SO_4 (96 – 98 %, ~ 7 mL) was added and the mixture was kept stirring at 0 °C on an ice bath. Potassium permanganate powder ($KMnO_4$, 900 mg) was added slowly (portion-wise) to the suspension. The addition rate was carefully controlled so that the temperature of the suspension was kept below 20 °C then stirred on an ice bath (0 – 10 °C) for 10 minutes. The ice bath was then removed and replaced by a water bath to allow the temperature to rise to 35 ± 3 °C, which was maintained for at least 30 minutes. Deionised water (DI H_2O) (14 mL) was then added slowly

into the pasty mixture. The temperature started to increase to ~ 98 °C with the appearance of a violet vapour. The mixture was kept stirring for 30 minutes then diluted with an additional 42 mL of DI H_2O. To stop the reaction, hydrogen peroxide (H_2O_2) aqueous solution (3 %, 50 mL) was added into the mixture and kept stirring for 10 minutes. H_2O_2 reduces the residual manganese dioxide (MnO_2) and permanganate (MnO) forming a brown suspension. The warm brown suspension was centrifuged at 3214 × g (3900 rpm) for 5 minutes (5810R, Eppendorf®, Germany). The supernatant was removed and the pellet was re-suspended in warm DI H_2O. The centrifugation and washing steps were repeated several times until the pH of the supernatant became neutral. The loosely packed GO flakes from the upper layer of the pellet were collected and re-suspended into water by gentle shaking. A yellow-brown GO suspension was produced. The concentration (w/v) of GO in the sample was determined by thermogravimetric analysis (TGA).

Preparation of GO by modified Kovtyukhova-Hummer's method

Modified Hummer's method reported by Kovtyukhova *et al.* was adapted with some modification.[2] Steps involved in preparation of GO are described in details below and schematically summarised in **Scheme S1**.

(i) Pre-oxidation of graphite: graphite powder (4 g), potassium persulfate ($K_2S_2O_8$, 2 g) powder, and phosphorus pentoxide (P_2O_5, 2 g) were dry-mixed in a 250 mL round bottom flask followed by the addition of sulfuric acid (H_2SO_4, 96 - 98%, 10 mL). The mixture was refluxed at 80 °C for 6 hr. The mixture was left to cool down to room temperature then diluted in DI H_2O (volume was not critical). A Millipore® All-Glass 47 mm Vacuum Filter Unit with hydrophilic polycarbonate Isopore™ membrane (0.22 μm) was then used to wash the mixture until the filtrate became neutral. The filter cake was oven dried at 40 °C for overnight affording a black-grey powder.

(ii) Low temperature stage: sodium nitrate powder ($NaNO_3$, 4 g) and dried pre-oxidised graphite powder (~ 4 g) and was dry-mixed in a 500 mL round bottom flask. Sulfuric acid (H_2SO_4, 96 - 98%, ~ 100 mL) was added and the mixture was kept stirring at 0 °C on an ice bath. When the powders were fully dispersed, potassium

permanganate powder ($KMnO_4$, 12 g) was added slowly to the suspension. The addition rate was carefully controlled so that the temperature of the suspension was kept below 20 °C. After the addition of $KMnO_4$ was complete, the suspension was stirred on an ice bath (0 - 10°C) for an additional 10 minutes.

(iii) Medium temperature stage: The ice bath was removed and replaced with a water bath to allow the temperature to rise gradually to 35 ± 3 °C. The temperature was maintained for at least 2 hr until the dispersion became a brown pasty mixture. The mixture was then heated to 60 °C.

(iv) High temperature stage: When the temperature reached 60 °C, 50 mL of DI H_2O was added to the pasty mixture and stirred for 5 minutes. The temperature of the oil bath was then set to 80 °C followed by the addition of 50 mL of DI H_2O. When the oil bath temperature reached 80 °C, 80 mL of DI H_2O was added to the mixture. A white vapour was generated. When the water addition was complete, the oil bath temperature was maintained above 80°C (temperature fluctuated between 80-95°C) and kept stirring for another 30 minutes. Hydrogen peroxide (H_2O_2) aqueous solution (35 %, 10 mL) was added to the mixture then kept stirring for 30 minutes at 80 °C to stop the reaction.

(v) Washing and purification: The warm brown suspension from the previous step was transferred into 10 × 50 mL conical-bottom centrifuge tubes and centrifuged at 3,214 × g (3900 rpm) for 2 minutes. The supernatant was removed and the pellets were resuspended in 1N HCl using a spatula with agitation. The centrifugation and washing steps were repeated several times with 1N HCl (1L) followed by 1N NaOH (0.5 ~ 1 L). The washing and centrifugation steps were monitored by observing the colour of the supernatant. The colour changed from yellow-green (early HCl washing stage), nearly transparent (late HCl washing stage), light brown (early NaOH washing stage) and finally to dark brown when NaOH washing was stopped. The pellets were resuspended in DI H_2O. Washing and centrifugation steps were repeated twice. The pellets were finally resuspended in water (50 mL/tube) and bath sonicated for 1 hr (USC300TH, VWR, Belgium). The sonicated mixture was centrifuged once at 3,214 × g (3900 rpm) for 2 minutes. GO suspension was obtained by strongly hand- shaking the centrifuge tubes and the supernatant was collected. Oxidised-graphite remained as pellet no matter how hard the shaking was. The collected supernatant was then diluted

with DI H_2O (volume was not critical) forming a dark-brown GO suspension. The final concentration of GO in the dispersion was determined by thermogravimetric analysis (TGA) as will described later.

Synthesis of GO-N_3

GO-N_3 was generated by reaction of GO dispersion (50 mL, 3 mg/mL) with sodium azide (600 mg, powder) in a 250 mL round bottom flask. The pH of the suspension was adjusted to 4.5 using 1% HCl and 1N NaOH solutions. The mixture was stirred overnight at room temperature (**Scheme 2**). When the reaction was complete, the mixture was transferred into a 50 mL centrifuge tube loaded with 20 mL CH_2Cl_2 and centrifuged at 3,214 × g (3900 rpm) for 2 minutes. GO-N_3 separated as a middle layer after centrifugation. The upper aqueous layer was discarded and the washing/centrifugation step with DI H_2O was repeated 10 times. CH_2Cl_2 was then discarded and finally GO-N_3 was resuspended in 50 mL DI H_2O. The final product (GO-N_3) was obtained by freeze-drying. Characterisation was performed using ATR-FTIR and Raman spectroscopy.

Qualitative assessment of azide group with Staudinger-Ninhydrin assay

In this study, samples (10 mg/each) were co-heated with 1 mL of freshly prepared 10% PPh_3 in CH_2Cl_2 (w/v) in a glass vial, at 80 °C for 30 minutes. The PPh_3 pre-treated samples and Ninhydrin (125 µl, 50 mg/mL EtOH) were co-heated in 3 mL 60 % EtOH at 100 °C for 7 minutes. The same experiment was repeated without the use of PPh_3 as negative controls. The qualitative comparison results were determined by visual inspection of the solution colour-change. The scheme describing this reaction is shown in **Scheme S3**.

Synthesis of $GO-N_3$ with epoxidised GO (epo-GO)

Direct epoxidation of GO with meta-chloroperoxybenzoic acid (mCPBA) has not been reported in the literature. In this work, mCPBA was used to introduce epoxides on GO surface to form epo-GO. Methods of epo-GO preparation is described below and summarised in **Scheme S4**.

In brief, GO suspension (50 mL, 3 mg/mL) and mCPBA <77 % (300 mg) were loaded into a round bottom flask, stirred for 24 hr at room temperature. As shown in **Figure S18**, mCPBA was firstly removed by extraction using CH_2Cl_2 (4 × 100 mL). The aqueous layer was then transferred into a 50 mL centrifuge tube loaded with 20 mL CH_2Cl_2 and centrifuged at 3,214 × g (3900 rpm) for 2 minutes. The upper aqueous layer was discarded and refilled with DI H_2O. The process was repeated for 10 times. CH_2Cl_2 was then removed; $GO-N_3$ was resuspended in 50 mL DI H_2O. The final product ($GO-N_3$) was obtained by freeze-drying and stored for subsequent characterisation using TGA and ATR-FTIR.

Synthesis of Click^2 GO (azide-, alkyne-double clickable GO)

In order to introduce azide and alkyne functional groups, a one-pot sequential synthesis strategy for azide and alkyne double functionalised GO (Click^2 GO) was attempted in this study. Reaction steps are summarised in **Scheme 1** and described as below:

(i) GO epoxidation: epo-GO was firstly prepared from GO (50 mL, 3mg/mL) using the mCPBA pre-treated method described in the previous section. However, after the reaction was complete, the entire suspension was then used directly for the next step without purification.

(ii) Azide introduction: NaN_3 (300 mg/each condition) was then mixed with epo-GO suspension. pH was adjusted to 4.5 using 1N NaOH and 1N HCl solutions. The mixture was stirred overnight at room temperature to afford mono-functionalised $GO-N_3$. The suspension was used directly for the next step without purification.

(iii) TMS-protected alkyne introduction: 3-(trimethylsilyl)propargyl alcohol (TMS-

protected progargyl alcohol, 400 µl, 2.7 mmol), 1-Ethyl-3-(3-dimethylaminopropyl)carbodiimide (EDC, 280 mg, 1.45 mmol) and 4-(Dimethylamino)pyridine (DMAP, 60 mg, 0.5 mmol) were mixed with the GO-N_3 suspension from previous step and stirred overnight at room temperature. This step was carried out to introduce the second functional group, the alkyne, onto GO-N_3 through Steglich esterification.[3] EDC was used for the coupling instead of N,N'-dicyclohexylcarbodiimide (DCC). The isourea by-product generated from EDC was water-soluble compared to water-insoluble dicyclohexylurea (DCU) from DCC so the former can be easily removed by water in the washing step.

(iv) Washing step: Reagents were removed by filtration. Reaction crude was washed with water (4 × 200 mL) followed by methanol (3 × 100 mL) and filtered using a Millipore® All-Glass 47 mm Vacuum Filter Unit and hydrophilic polycarbonate Isopore™ membrane (0.22 µm). Precipitant on the filter was collected, freeze-dried and stored for further characterisation.

Synthesis of di-azo transferring reagent: imidazole-1-sulfonyl azide hydrochloride

Imidazole-1-sulfonyl azide hydrochloride was synthesised according to the method reported in the literature.[4] In brief, sulfuryl chloride (SO_2Cl_2; 16.1 mL, 200 mol) was added drop-wise to an ice-cooled suspension of NaN_3 (13.0 g, 200 mmol) in MeCN (200 mL). The mixture was stirred for an overnight at room temperature. Imidazole (25.9 g, 380 mmol) was added (portion-wise) to the ice-cooled dispersion forming white coloured slurry and stirred for 3 h at room temperature after the addition. The mixture was then made up to 350 mL using EtOAc, washed with deionised water (3 × 150 mL). The collected organic layer was made up to 300 mL using EtOAc then washed with saturated aqueous $NaHCO_3$ (3 × 150 mL), dried over $MgSO_4$ and filtered. A solution of HCl in EtOH was prepared by the drop-wise addition of acetyl chloride (AcCl/CH_3COCl; 21.3 mL, 300 mmol) to ice-cooled dry ethanol (75 mL). This was then added drop-wise to the filtrate with stirring to crystallise the compound. The highly exothermic crystallisation was performed slowly and chilled on an ice-bath. The suspension was then filtered. The filter cake was washed with EtOAc (3 ×

100 mL) to give imidazole-1-sulfonyl azide hydrochloride as colourless needles (yield: 18.03 g, 86mmol, 43%). IR ν_{max} (ATR) cm^{-1}: 2170 (N_3), 1914, 1582, 1509, 1426, 1322, 1298, 1160, and 776. ^1H NMR (400 MHz, D_2O): δ = 9.35 (dd, J = 3.8 Hz, 1.6 Hz, 1 H, H-2), 7.99 (m, 1 H, H-5), 7.59 (dd, J = 2.6 Hz, 2.0 Hz, 1 H, H-4). The reaction scheme, IR and ^1H NMR results are summarised in **Figure S8A**.

Synthesis of N_3-PEG_{3500}-N_3

NH_2-PEG_{3500}-NH_2 (700 mg, 0.2 mmol), imidazole-1-sulfonyl azide hydrochloride (418 mg, 2 mmol), K_2CO_3 (220 mg, 1.6 mmol), and $CuSO_4.5H_2O$ (1 mg, 4 μmol) were dissolved in MeOH (10 mL) and stirred for ~24 hr. The reaction was monitored using ninhydrin assay. The reaction was stopped when the ninhydrin test was negative. MeOH was removed *in vacuo*. The residual was dissolved in CH_2Cl_2 (100 mL) and extracted with water (3 × 100 mL) and saturated NaCl solution (1 × 100 mL). The organic phase was dried over $MgSO_4$, filtered, concentrated, then ice-cold Et_2O was added to precipitate the PEG. The PEG was collected by filtration, dissolved in CH_2Cl_2 and re-precipitated in ice-cold Et_2O. The final product was collected by filtration then air-dried in the fume hood for 48 hr. (Yield = 425.3 mg, 60%) The reaction scheme, IR and ^1H NMR results are summarised in **Figure S8B**.

Synthesis of propargyl-modified Angiopep-2

A modified Angiopep-2 sequence (TFFYGGSRGKRNNFKTEEYG) was synthesised using the CEM Liberty 1 microwave peptide synthesizer (CEM Microwave Technology Ltd, UK) on NovaSyn® TGR resin (0.5 mmol/g loading). The ε-amino group of lysine 15 (K^{15}) was protected using (4,4-dimethyl-2,6-dioxocyclohex-1-ylidene)ethyl (Dde). Propargylglycine was incorporated at G^{20} for click chemistry application on GO-N_3. Fmoc groups were deprotected with 20% piperidine in dimethylformamide (DMF) v/v (10 min x 2). Acylation cycles were carried out by mixing each of the amino acids with O-(6-Chlorobenzotriazol-1-yl)-N,N,N′ ,N′ -tetramethyluronium hexafluorophosphate (HCTU) (0.78 mmol) in the presence of

2,3,5-collidine (1.6 mmol) in 3 ml of DMF anhydrous. The mixture was incubated with the solid resin for 10 min using a microwave power of 25 W. The Dde group on the ε-amino group of K^{15} was selectively de-protected using a solution of hydrazine hydrate in DMF (2% v/v). The free ε-amino group on K15 was acylated with Fmoc-Cys(Trt)-OH residue (70 mg, 119 μmol), HCTU (0.78 mmol), and 2,3,5-collidine (1.6 mmol) in 3 ml of DMF anhydrous for 1 hr at RT. The synthesised peptide was cleaved from the solid resin and simultaneously deprotected using trifluoroacetic acid (TFA), water, phenol, triisopropylsilane, thioanisole and 2,2′(Ethylenedioxy)diethanethiol (77.5/5/5/5/5/2.5) for 3 hours. The cleaved peptide was precipitated in cold diethyl ether and washed three times with centrifugation (3220 *g*). The solid pellet was dissolved in deionised water and lyophilised. The molecular weight of the modified Angiopep-2 was confirmed by MALDI-TOF MS (Bruker, USA), using α-cyano-4-hydroxycinnamic acid, as the matrix. The monoisotopic mass of 2499 m/z matched the theoretical value. The chemical structure, IR spectrum, and MALDI-TOF-MS results for the crude peptide are summarized in **Figure S9**.

Double functionalisation of Click^2 GO

As-synthesised Click^2 GO was further functionalised with propargyl-modified angiopep-2 and N_3-PEG_{3500}-N_3 using two sequential CuAAC clicks **(Scheme 1 and S5)**. In brief, Click^2 GO (30 mg, 5.28 μmol of azide), propargyl-modified Angiopep (ANG, 13.2 mg, 5.8 μmol), L-ascorbic acid (9 mg), and $CuSO_4.5H_2O$ (7.9 mg) were dissolved/dispersed in 5 mL of 10% MeCN in H_2O. The mixture was stirred at room temperature for 24 hr then washed with 1 L H_2O by filtration to afford ANG-functionalised Click^2 GO as black precipitate (ANG-GO). The control click reaction was carried out without including ANG in the reaction, at 50% scale size. ANG-GO was re-dispersed into 24 mL H_2O and equally divided into 3 × 8 mL portions. The first portion was kept for ATR-FTIR and TGA analysis. The second portion was mixed with N_3-PEG_{3500}-N_3 (12 mg) for 48 hr then washed/filtered using 2L of 10% MeOH in H_2O (2nd click control). The third portion was reacted with N_3-PEG_{3500}-N_3 (12 mg), L-ascorbic acid (3 mg), and $CuSO_4.5H_2O$ (3 mg) for 48 hr, wash with 2L of 10% MeOH in H_2O then filtrated to afford ANG-GO-PEG. All compounds were freeze-dried and characterised by ATR-FTIR and TGA **(Figure 3** and **S10)**.

Physicochemical characterisation methods

Thermogravimetric analysis (TGA)

Liquid samples *e.g.* GO aqueous dispersions (75 µl) were firstly loaded on platinum sampling pans, dried at 120 °C for 2-minute. The concentration of the aqueous GO dispersions were calculated based on the residual weight after drying and the volume of loaded samples was expressed by the following equation:

$$Concentration\ (mg/mL) = residual\ weight\ (mg) \times \frac{75\ \mu L}{1000\ \mu L/mL} \times 100\% \qquad \textbf{Eq. S1}$$

To monitor the degree of chemical modification on the sample, dried aqueous or powder samples were then equilibrated at 100 °C for 10 minutes before heating up with a ramp of 10 °C /min from 100 to 1000°C under nitrogen with a flow rate of 90 mL/min using TGA model TGA-Q500 (TA instrument, USA). The TGA thermal curve is displayed as the weight (mg) or residual weight percentage (%) as a function of the temperature (°C) within the defined range.

Attenuated Total Reflectance Fourier Transform Infrared (ATR- FTIR)

ATR-FTIR was performed using PerkinElmer® Frontier™ FT- IR equipped with ATR accessory (diamond ATR polarization accessory with 1 reflection top-plate and pressure arm). The pressure arm was used for all solid samples at a force gauge setting between 100 ~ 120 units; no compression was used for liquid/oil samples. The number of scans was set at 15. Samples were loaded on the reflection top-plate at a quantity sufficient enough to cover the entire diamond surface. GO derivatives were dispersed in a suitable solvent (usually water for hydrophilic samples) and freeze dried into a loose sponge-like structure. The container was sealed immediately after freeze-drying. Moisture absorption was minimised when warming to room temperature in order to obtain a good spectrum resolution with minimum interferences.

Raman spectroscopy

Raman spectroscopy was performed using Renishaw® inVia-Reflex spectrometer (UK) with an excitation wavelength set to 785 nm and 0.1 to 1 % laser power. Aqueous dispersion of GO derivatives were deposited and dried on a calcium fluoride (CaF_2) slide (Crystran Ltd, UK) on a hot plate before the measurement. Measurements were performed from 500 cm^{-1} to 3200 cm^{-1} for each sample (n = 3). Data were acquired and analysed using Renishaw's WiRE 4.0 (Windows-based Raman Environment) software.

Atomic force microscopy (AFM)

The surface topography of GO samples deposited on a dry mica surface was studied by AFM using tapping mode. GO samples were prepared at a concentration of 0.2 mg/mL in water, deposited on the mica for 3 ~ 5 minutes. The more hydrophobic sample required longer deposition time. Excess samples were removed and the mica was dried under a gentle flow of compressed nitrogen (or a dust-free compressed air spray). The sampling was achieved by oscillating the tapping probe to hit the sample surface, which allowed short-time interactions with minimal shear force applied on the surface. The oscillation was analysed by collecting reflected laser spot signal from the probe cantilever (general purpose tips cat: NSC15/AL, MIKROMASCH, USA) with ScanAsyst® Dimension Icon® AFM (Bruker, UK).

Transmission electron microscopy (TEM)

TEM was performed on Philips CM 12 (FEI Electron Optics, The Netherlands) equipped with Tungsten filament and a Veleta - 2k × 2k side-mounted TEM CCD Camera (Olympus, Japan). The accelerating voltage is 80 KV. The spot size was set at 3. Objective aperture was used with all samples. GO aqueous dispersions at a concentration of 0.2 mg/mL were deposited on carbon-film on 300 mesh copper grids or lacey carbon films on 300 mesh copper grids for the measurement.

GO flake surface area analysis

The surface area of the GO flake was analysed using Image J 1.49i software from National Institute of Health (USA). Images from AFM were used for analysis with a

total measurement number no less than 100 flakes for each sample. Histogram of surface area distribution and statistical calculation was generated and performed using DataGraph 3.2 (Visual Data Tools, Inc., USA).

Elemental analysis and GO yield calculation

Elemental analysis of samples was performed by Medac Ltd., UK (*n* = 2) using FlashEA® 1112 Elemental Analyzer (CHNS-O). Samples were placed in a tin capsule and oxidised instantantly by flash combustion at 1800 °C in helium gas (He) that temporary enriched with pure oxygen gas (O_2). All organic and inorganic substances were converted into combustion products, which were then passed through a catalyst layer with copper to remove excess oxygen and to reduce the nitrogen oxide to elemental nitrogen (N_2). Samples were separated and eluted as N_2, carbon dioxide (CO_2), water (H_2O) and sulphur dioxide (SO_2). The analysis was performed using gas chromatographic (EAGER300™ workstation) with a thermal conductivity detector (TCD). C, H, N % were measured as % atom. O % was calculated by subtraction assuming that the sample consisted only of C, H, N and O. C, H, N and O % were calculated as percentage weight of the total sample (w/w %) using Eq. S2. GO yield was calculated based on C % (w/w %) content using Eq. S3.

$$Element\ Wt.\% = \frac{Element\ \% \times Atomic\ Mass}{(C\% \times 12) + (H\% \times 1) + (N\% \times 14) + (O\% \times 16)} \times 100\% \qquad \textbf{Eq. S2}$$

$$GO\ Yield\ \% = \frac{Carbon\ Wt.\% \times GO\ Weight\ (mg)}{Starting\ Graphite\ Weight\ (mg)} \qquad \textbf{Eq. S3}$$

Statistical analysis

The data were expressed as mean ± standard deviation (mean ± S.D.). All the statistical analyses were implemented using Minitab® v16 (Minitab Inc., UK). The comparison between the control and experiment groups was analysed using student t-

test of one-way ANOVA followed Tukey's HSD (honest significant difference) tests. Mean differences with $p < 0.05$ were considered significant.

GO characterization

Thermogravimetric analysis (TGA) was performed to analyse the thermal-decomposition profile of graphite, pre-oxidised graphite and GO (100–978 °C). As shown in **Fig. S2A**, the thermal deoxygenation of graphene derivatives was reflected by the reduced residual weight upon heating (101°C min^{-1} in nitrogen). The final residual weight at 978 1C was 99.36%, 94.15% and 38.16% for graphite, pre-oxidised graphite and GO, respectively. Raman spectroscopy confirmed the generation of GO (**Fig. S2B**). D, G and 2D peaks were identified for both graphite and GO. The spectra were normalised to G peak (intensity equals 1). G peak at 1580 cm^{-1} (graphite) or 1598 cm^{-1} (GO) represent the bond stretching of all pairs of sp^2 atoms (double bonds) in both rings and chains. It is used to represent the integrity of the organised graphitic structure. The increased wavenumber of the G peak in GO (by 18 cm^{-1}) indicated an increased sp^3 content (single bonds i.e. defects) when compared to graphite. The D peak at 1 1324 cm^{-1} (graphite) or 1326 cm^{-1} (GO) is attributed to the breathing modes of sp^2 atoms in the ring and is usually activated by defects present on the graphitic surface. The respective I_D/I_G (peak height) values of graphite and GO were 0.21 ± 0.01 and 1.22 ± 0.01, 5 respectively (n = 3). Higher I_D/I_G value indicated an increased disorder of the graphitic structure, thus being more defective. The relative thickness (number of layers) of the sheet was reflected by the lower intensity of 2D peaks at 2613 cm^{-1} for GO (few layers)and higher 2D intensity at 2649 cm^{-1} for graphite (410 layers). Morphological examination of GO was further studied by TEM andAFM as shown in **Fig. S3-4**. GO suspension appeared to consist mainly of large and multi-layered GO sheets.

Biological methods

The modified Lactate dehydrogenase (LDH) assay for A549 cells

A549 cells were maintained in DMEM containing 4.5 g/L glucose, supplemented with 10 % heat-inactivated FBS, 100 U/mL penicillin, 100 μg/mL streptomycin, 10 mM HEPES, 2 mM GlutaMax, 1 mM sodium piruvate and cultured at 37 °C under a humidified atmosphere containing 5 % CO_2. A549 cells were seeded onto a 96-well plate (10^4 cells per well) and allowed to divide for 24 hr. GO dispersions, prepared in 5 % dextrose solution, were then added onto the cells at a final concentration of 10, 50 or 100 μg/ml for 24 and 72 hr. The cells were then washed three times with HBSS and lysed upon incubation for 1 hour at 37 ºC with Triton X-100 (0.9 % v/v in fresh phenol-free DMEM). The cell lysates were subsequently centrifuged for 2 hr at 4000 rpm (Eppendorf 5810R, Germany). The LDH activity was measured in the supernatant using the CytoTox 96® assay protocol (Promega Corporation, USA), following the manufacturer's instructions, and the absorbance at 490 nm was measured in a FLUOstar Omega microplate reader (BMG Labtech, Germany). Cell viability was calculated as percentage of control untreated cells using the equation: (A_{490} of treated cells - A_{490} of negative control) / (A_{490} of untreated cells - A_{490} of negative control) × 100 %.

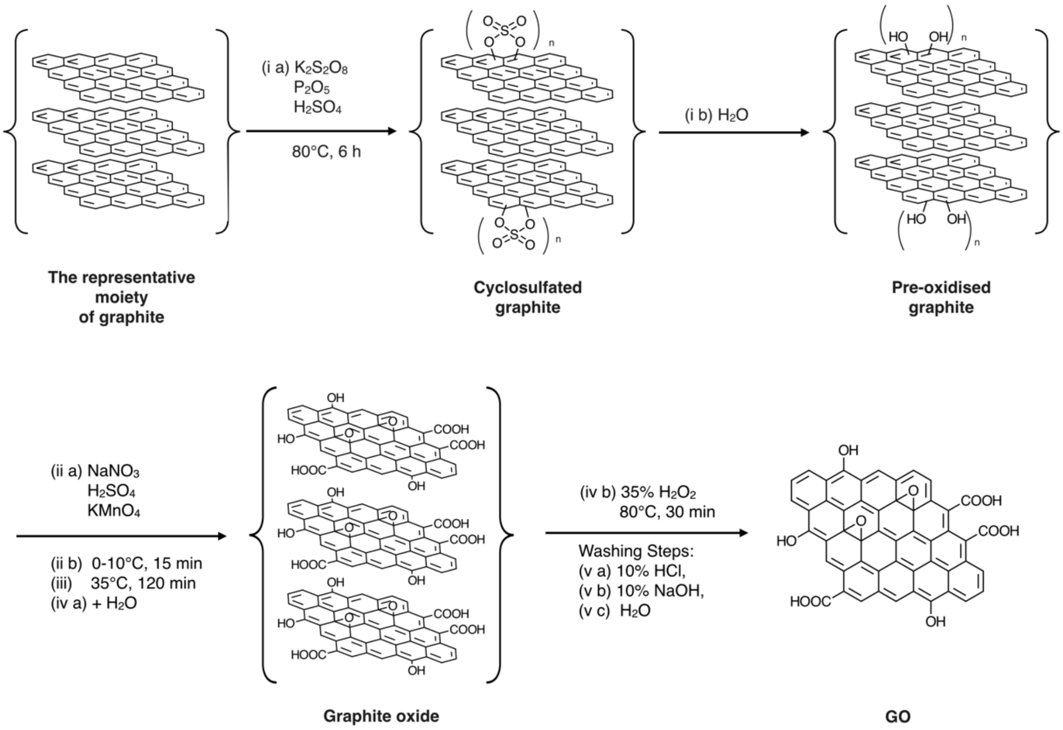

Scheme S1: Preparation of GO by modified Kovtyukhova-Hummer's method.

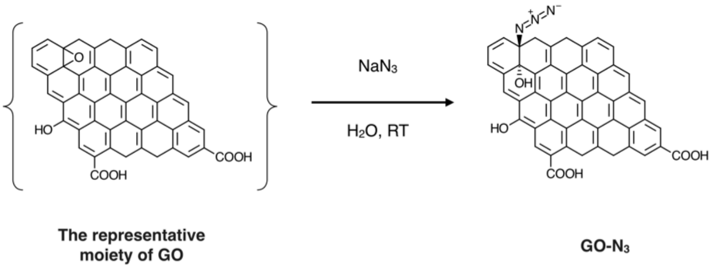

Scheme S2: Synthesis of GO-N_3 *via* 1,2-epoxide nucleophilic ring-opening.

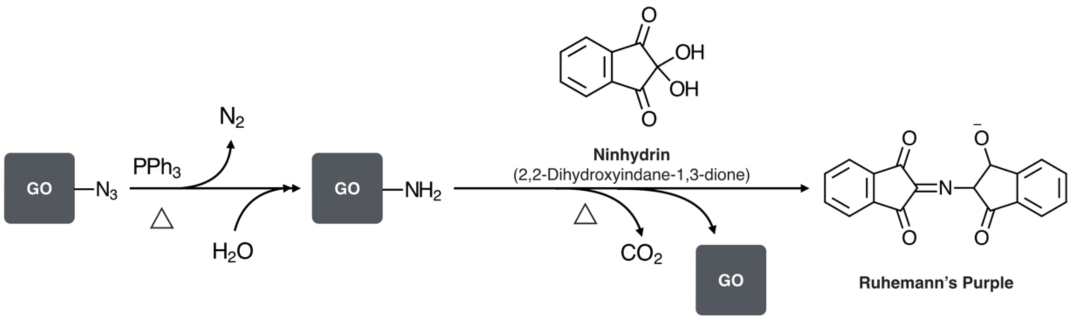

Scheme S3: Staudinger-Ninhydrin reaction used for azide group detection on GO.

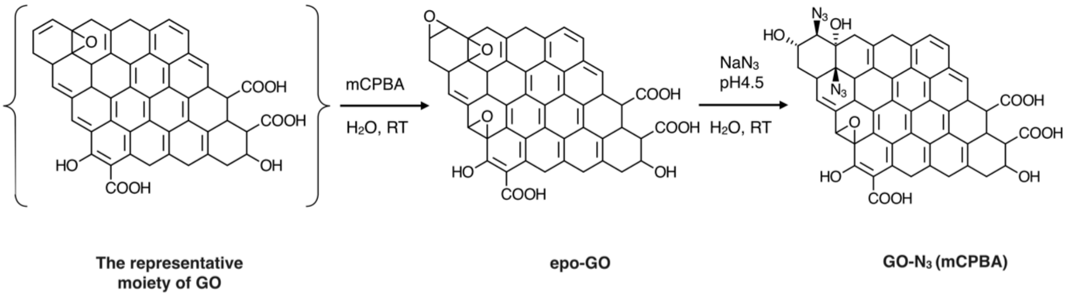

Scheme S4: GO-N_3 synthesised from epoxidised GO.

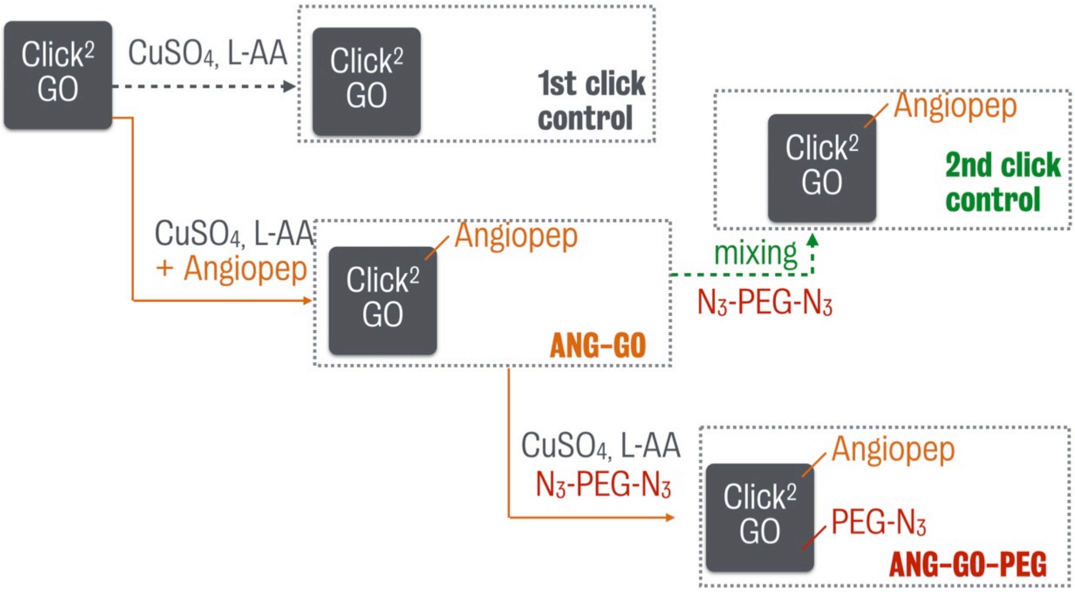

Scheme S5. Experimental design for sequential Click^2 functionalisation.

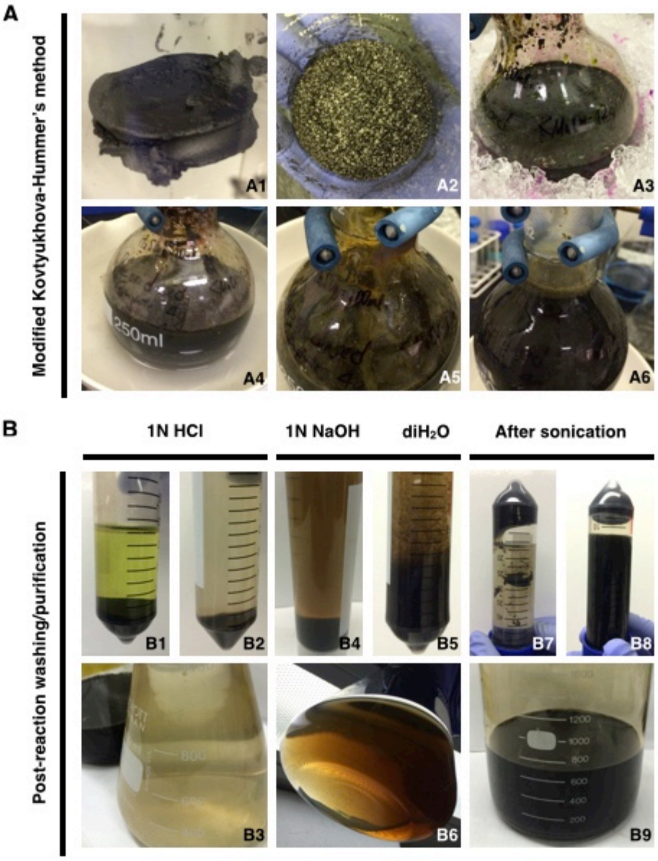

Figure S1: Preparation of GO using modified Kovtyukhova-Hummer's method. Graphite was firstly oxidised by P_2O_5 and $K_2S_2O_8$ at 80 °C for 6 hr then washed by filtration with deionised water. The washing was stopped when the pH of the filtrate became neutral. The filter cake was collected **(A1)** and dried overnight in an oven to obtain pre-oxidised graphite powder **(A2).** Pre-oxidised graphite was further oxidised with $NaNO_3$ and $KMnO_4$ in H_2SO_4 at low temperature **(A3)**, mid-temperature (35°C, 2 hr) **(A4)**, then heated the oil bath to 60 °C to start the hydration stage at higher temperature **(A5).** The oil bath temperature was maintained >80°C for 30 minutes. 35% H_2O_2 was added and kept stirring at 80°C for 30 minutes to terminate the reaction **(A6).** One litre of 1N HCl was used to wash the mixture and removed by centrifugation. The colour of the supernatant was yellow-green at the beginning **(B1)** then turned into almost transparent at the end **(B2).** The 1N HCl washing waste was collected and discarded **(B3).** Roughly 500 mL 1N NaOH was used to wash the mixture by centrifugation. The washing was stopped when the supernatant turned brownish **(B4).** DI H_2O was used to wash the mixture (2X). Floating aggregates (part of the GO) were found in the supernatant after centrifugation **(B5).** The brownish NaOH/DI H_2O washing waste was collected and discarded **(B6).** The pellet was resuspended in DI H_2O, sonicated for 1 hour to exfoliate the GO from graphite oxide. The mixture was centrifuged after the sonication. A loosely packed GO cake was formed on the upper layer of the pellet. The cake was separated by turning the centrifuge tube upside down **(B7)** and resuspended by hand shaking **(B8).** The collected GO suspension was then combined and stored at room temperature in a glass bottle before use **(B9).**

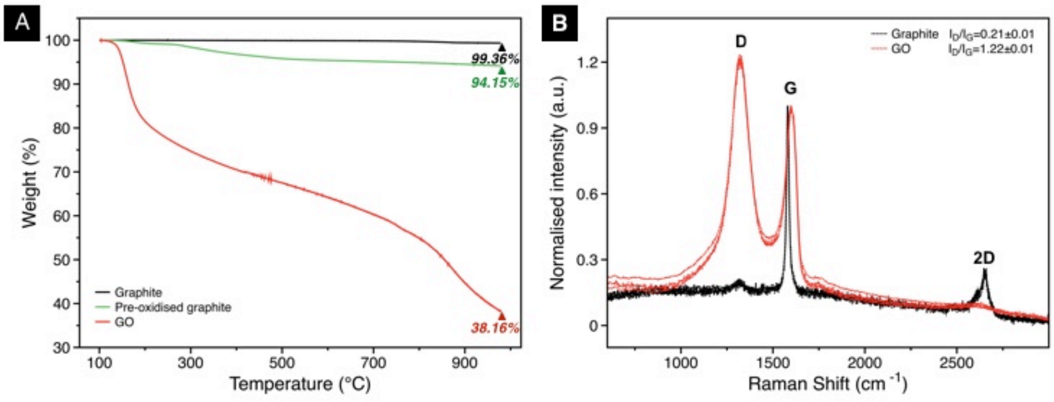

Figure S2: Physicochemical analysis of GO. (A) TGA thermal-curve of graphite (black), pre-oxidised graphite (green) and GO (red) ranging from 100 − 978 °C. The thermal deoxygenation of graphene derivatives was reflected by the reduced residual weight upon heating (10 °C /min in nitrogen). The final residual weight at 978 ℃ was 99.36 (graphite), 94.15 (pre-oxidised graphite) and 38.16 % (GO). **(B)** Comparison of the Raman spectra of graphite (black) and GO (red) measured at 785 nm is shown. The G peaks at 1580 cm^{-1} (graphite) and 1598 cm^{-1} (GO) represent the bond stretching of all pairs of sp^2 atoms in both rings and chains. The D peaks at 1324 cm^{-1} (graphite) and 1326 cm^{-1} (GO) are attributed to the breathing modes of sp^2 atoms in ring that are usually activated by the defect on the graphitic surface. The I_D/I_G ratios of graphite and GO were 0.21 ± 0.01 and 1.22 ± 0.01 respectively (*n* = 3). The relative thickness (number of layers) of the sheet was reflected by the intensity of 2D peaks at 2613 cm^{-1} (GO, few layers) and 2649 cm^{-1} (graphite, > 10 layers).

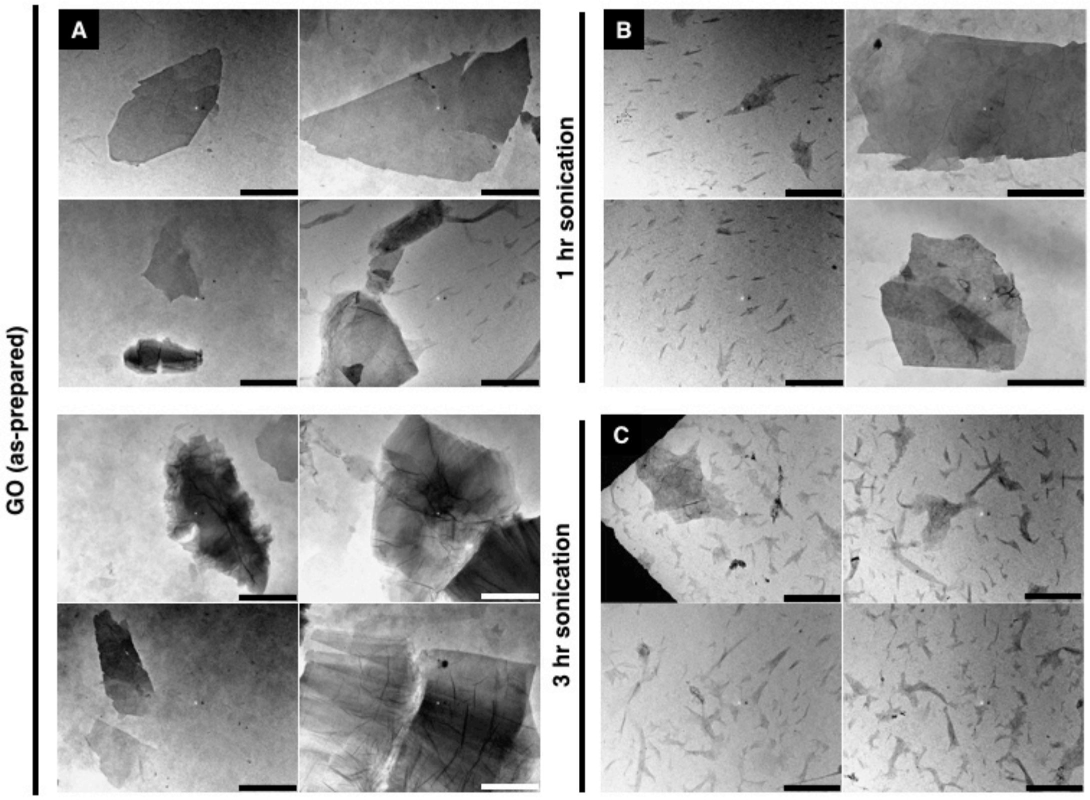

Figure S3: TEM images of GO with different sonication time. TEM images of the as prepared GO stock suspension produced by the modified Kovtyukhova-Hummer's method without subsequent sonication **(A)** or sonicated for an additional 1 hr **(B)** or 3 hr **(C)**. Scale bar is 5 μm. The stock suspension appears to consist mainly of large and multi-layered GO sheets but smaller GO sheets were also found. GO flakes size (surface area) was reduced after 3 hr of sonication. The majority of sonicated GO appeared to be more transparent (less layers) than the as prepared GO (stock suspension).

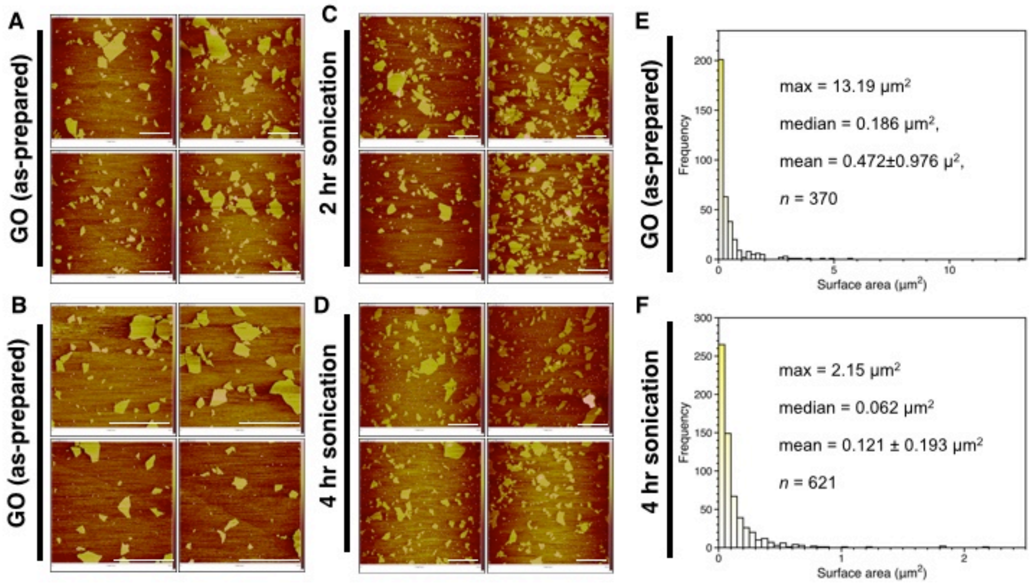

Figure S4: AFM height images of GO with different sonication times and flake surface area analysis. AFM images of the as prepared GO stock suspension produced by the modified Kovtyukhova-Hummer's method without subsequent sonication **(A, B)** or sonicated for an additional 2 hr **(C)** or 4 hr **(D)**. Scale bar = 5 µm. GO flake size (surface area) was reduced after 2 and 4 hr of sonication. The flake surface area analysis for GO **(E)** and GO after 4 hr sonication **(F)** showed a reduced max, median, mean surface area, and S.D. after prolonged sonication.

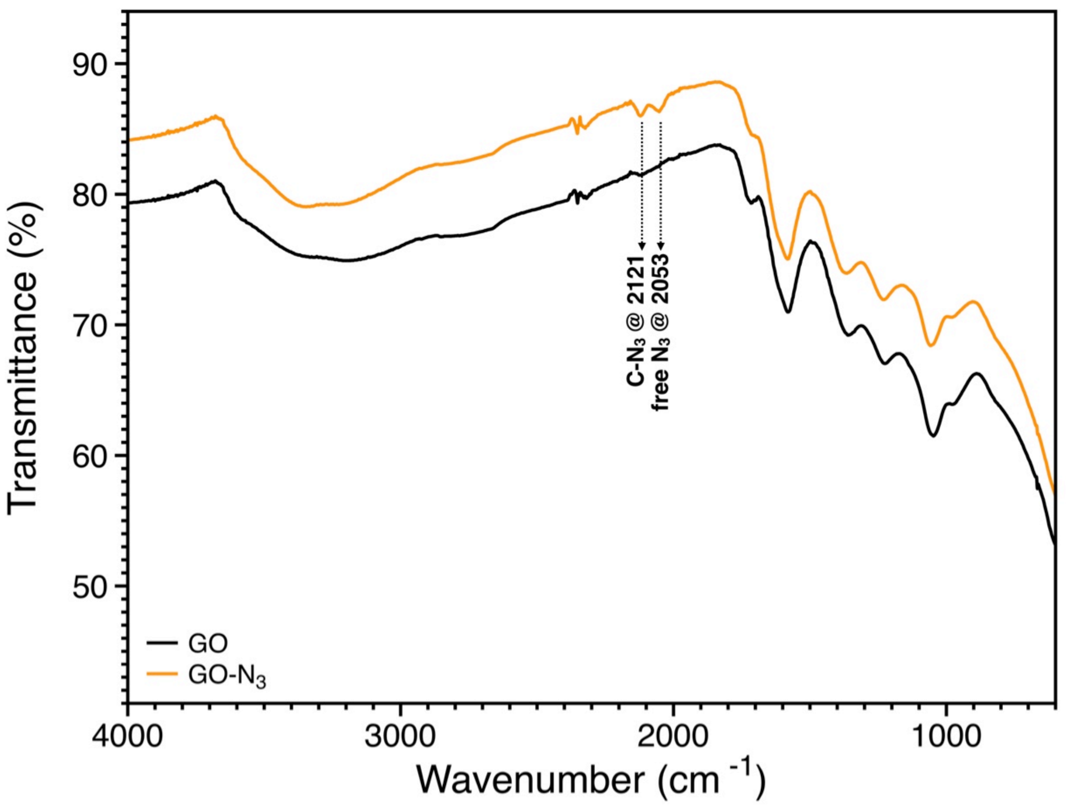

Figure S5. Infrared-transmittance Spectrum for GO-N_3 with free azide impurities.

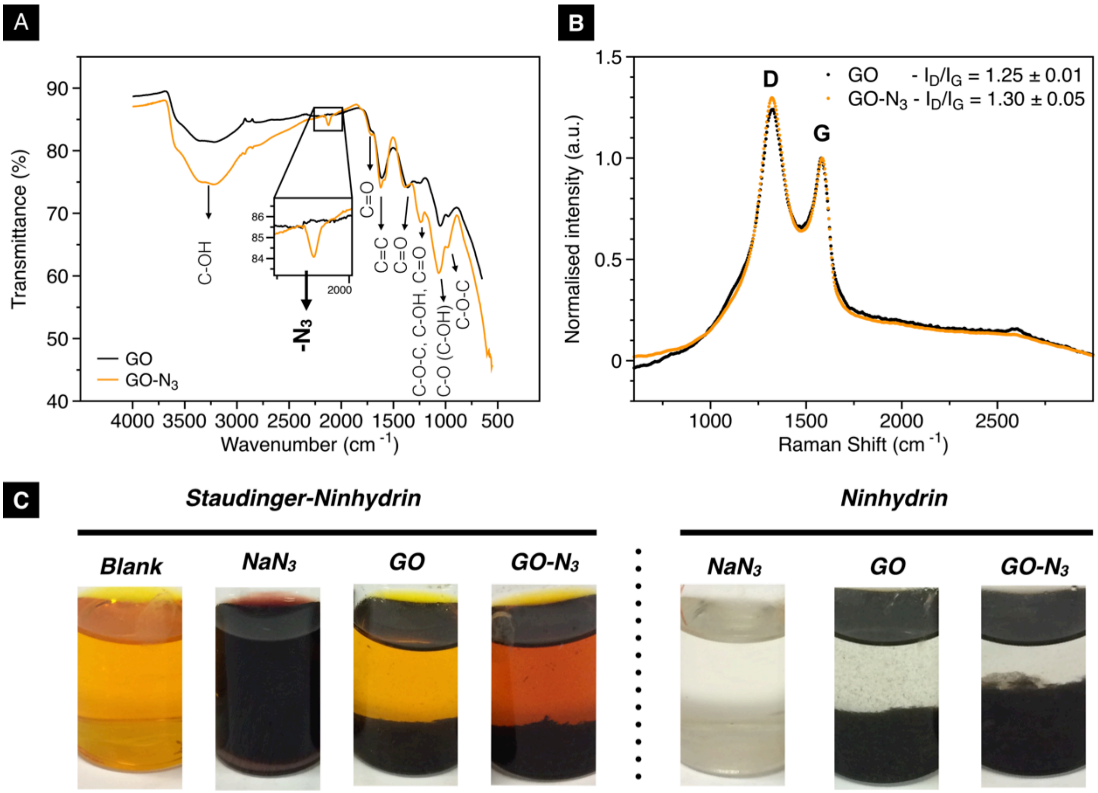

Figure S6. Physicochemical Characterisation of GO-N_3. (A) Infrared-transmittance Spectrum for GO-N_3. The IR spectra of GO (black) and GO-N_3 (orange) are shown. The presence of different oxygen functionalities was confirmed for both samples at 3200 cm^{-1} (broad bonded O-H stretching vibration), 1700 cm^{-1} (carbonyl C=O stretching vibration), 1605 cm^{-1} (aromatic C=C stretching vibration), 1356 cm^{-1} (carboxyl O-C=O stretching vibration), 1227 cm^{-1} (symmetric stretching of epoxy groups, C-OH, or ketone), 1036 cm^{-1} (C-O in C-OH or C-O-C) and 971 cm^{-1} (C-O-C stretching vibration). The introduction of azide groups was observed by the presence of azide peak (-N_3) at 2122 cm^{-1}. **(B)** Raman spectra of GO (black) and GO-N_3 (orange) measured at 785 nm are shown. The I_D/I_G ratios of GO and GO-N_3 were 1.25 ± 0.01 and 1.30 ± 0.05, respectively (n = 3). **(C)** Both Studinger-Ninhydrin (PPh_3/Ninhydrin treated) and traditional Ninhydrin (Ninhydrin treated) assays were used to verify the presence of azide groups. Photographs at day 0 and day 9 are shown. Ninhydrin assay showed negative results (colourless supernatants) for NaN_3, GO and GO-N_3. Staudinger-Ninhydrin assay showed positive results (orange-red supernatant) for both NaN_3 (positive control) and GO-N_3 samples, while GO showed negative result (light orange supernatant) as that shown for the blank (all reagents except azide containing compound).

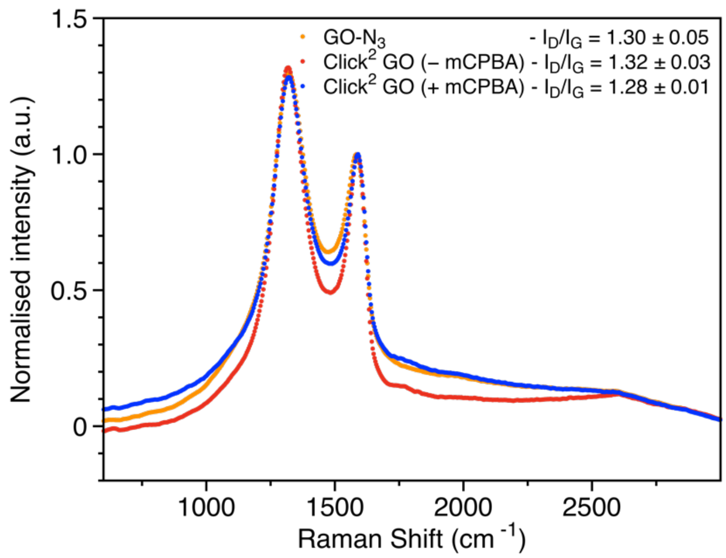

Figure S7. Raman Spectra of GO-N_3, Click^2 GO (± mCPBA). Raman spectra of GO-N_3 (orange) and Click^2 GO (- mCPBA) (red) and Click^2 GO (+ mCPBA) (blue) measured at 785 nm are shown. The I_D/I_G ratios of GO-N_3, Click^2 GO (- mCPBA) and Click^2 GO (+ mCPBA) were 1.30 ± 0.05, 1.32 ± 0.03, and 1.28 ± 0.01 respectively (n = 3).

A

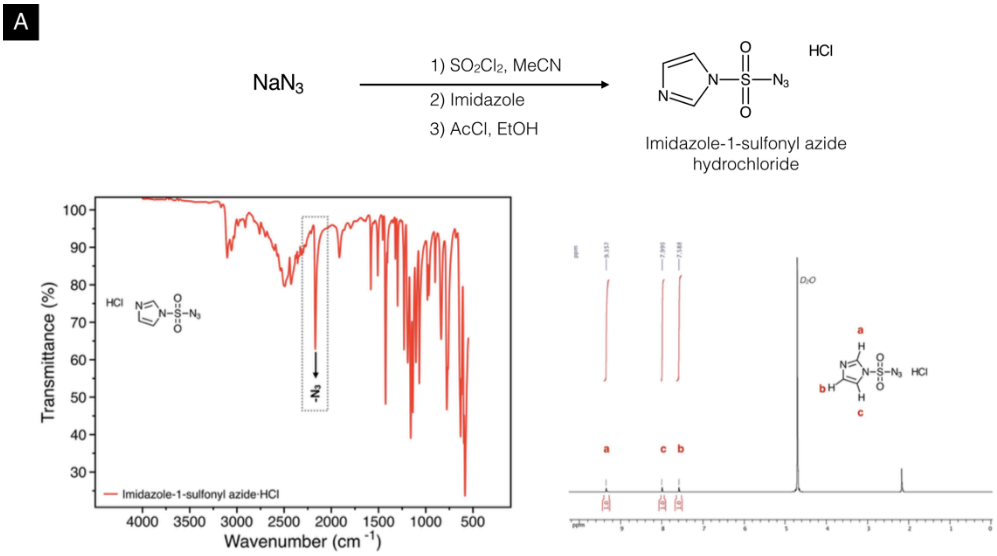

B

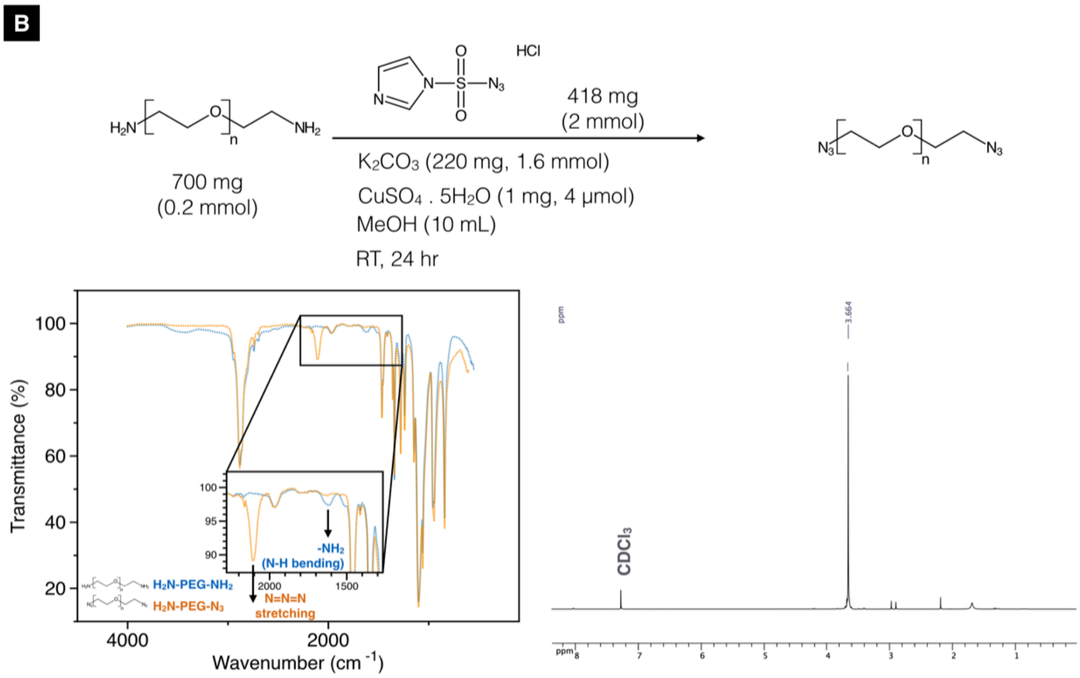

Figure S8. Synthetic scheme, FT-IR and ^{1}H-NMR of (A) imidazole-1-solfonyl azide hydrochloride and (B) N_3-PEG_{3500}-N_3.

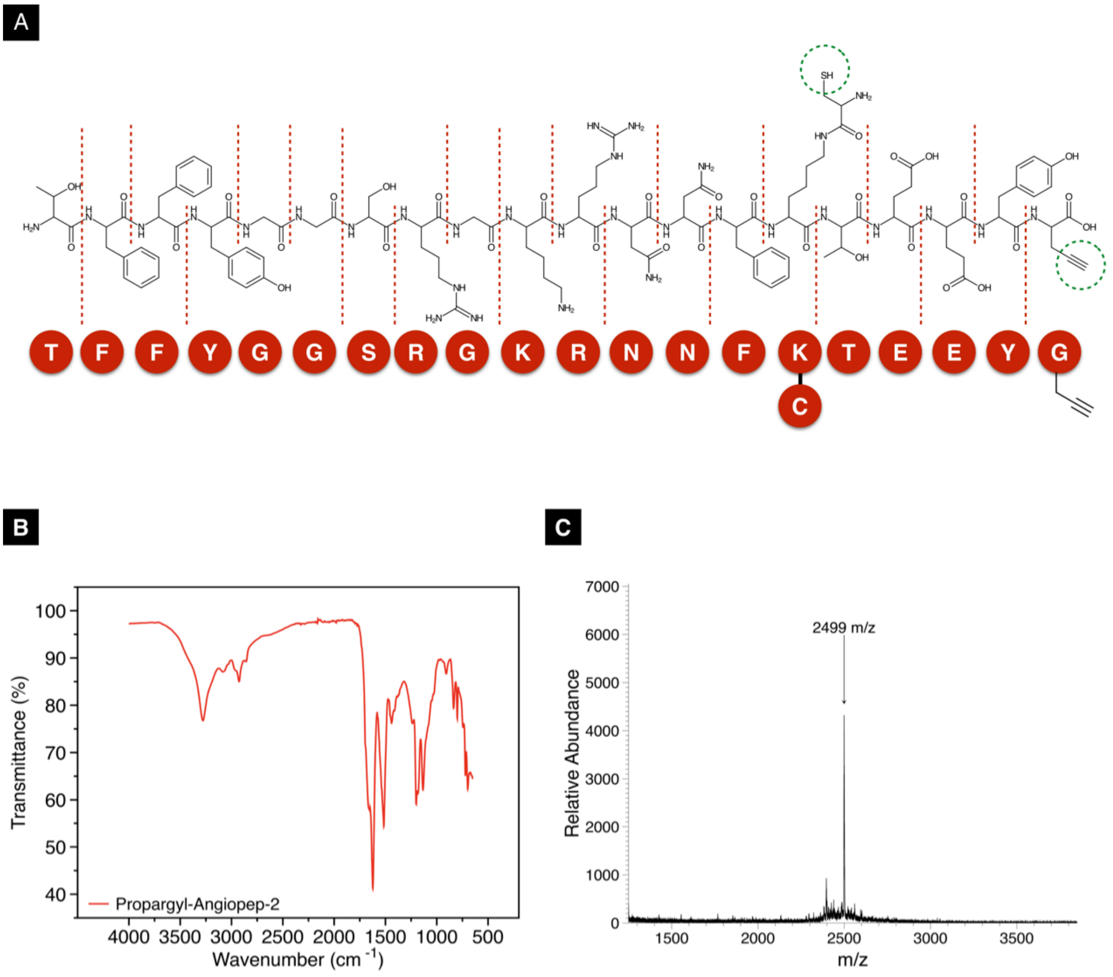

Figure S9. SPPS of propargyl-modified angiopep-2. (A) Amino acid sequence, (B) FT-IR and (C) matrix-assisted laser desorption/ionization time of flight mass spectrometry results (MALDI-TOF MS) of crude Angiopep-2.

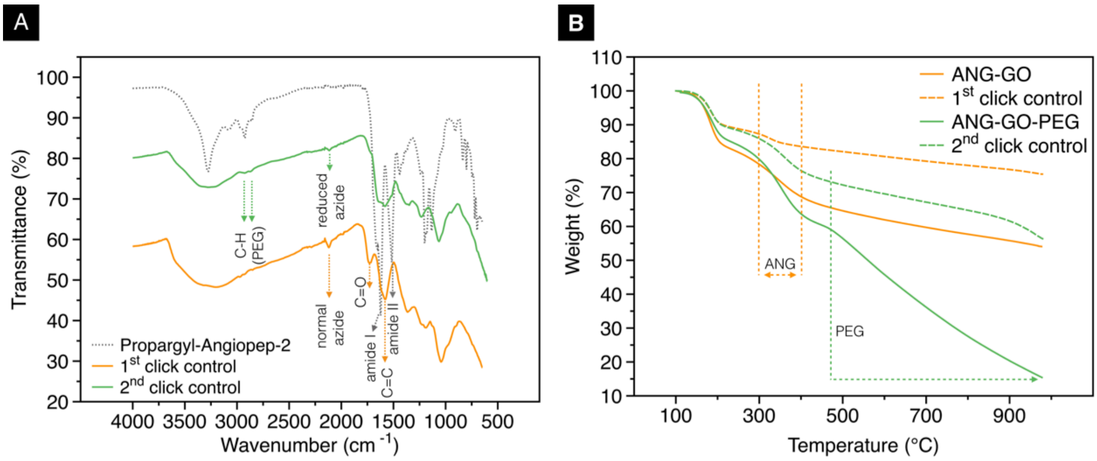

Figure S10. (A) Infrared-transmittance spectra and (B) thermogravimetric analysis of click reaction controls.

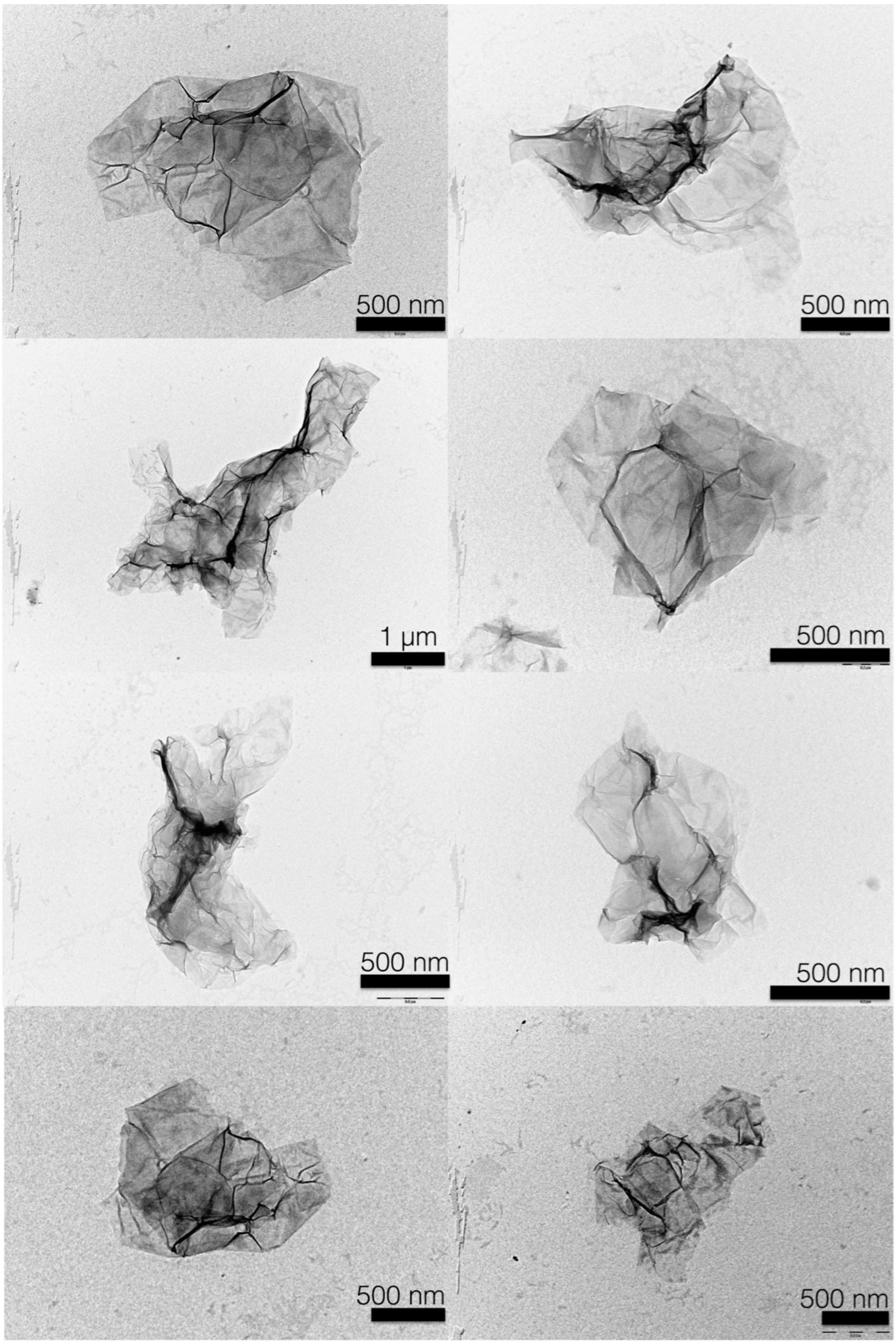

Figure S11: TEM images of GO clicked with Angiopep-2 (ANG-GO).

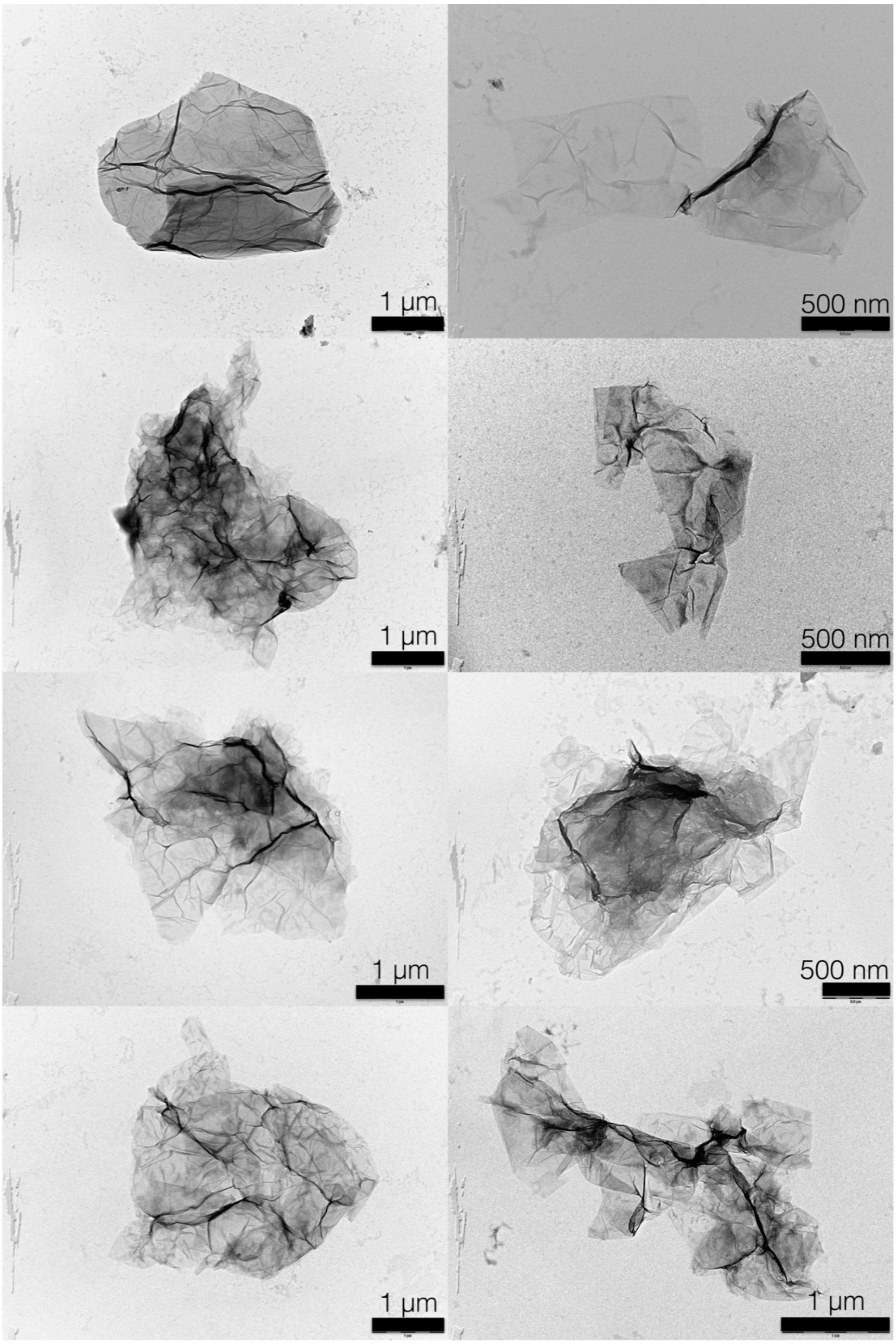

Figure S12: TEM images of GO double-clicked with alkyne modified Angiopep-2 and N_3-PEG-N_3 (ANG-GO-PEG).

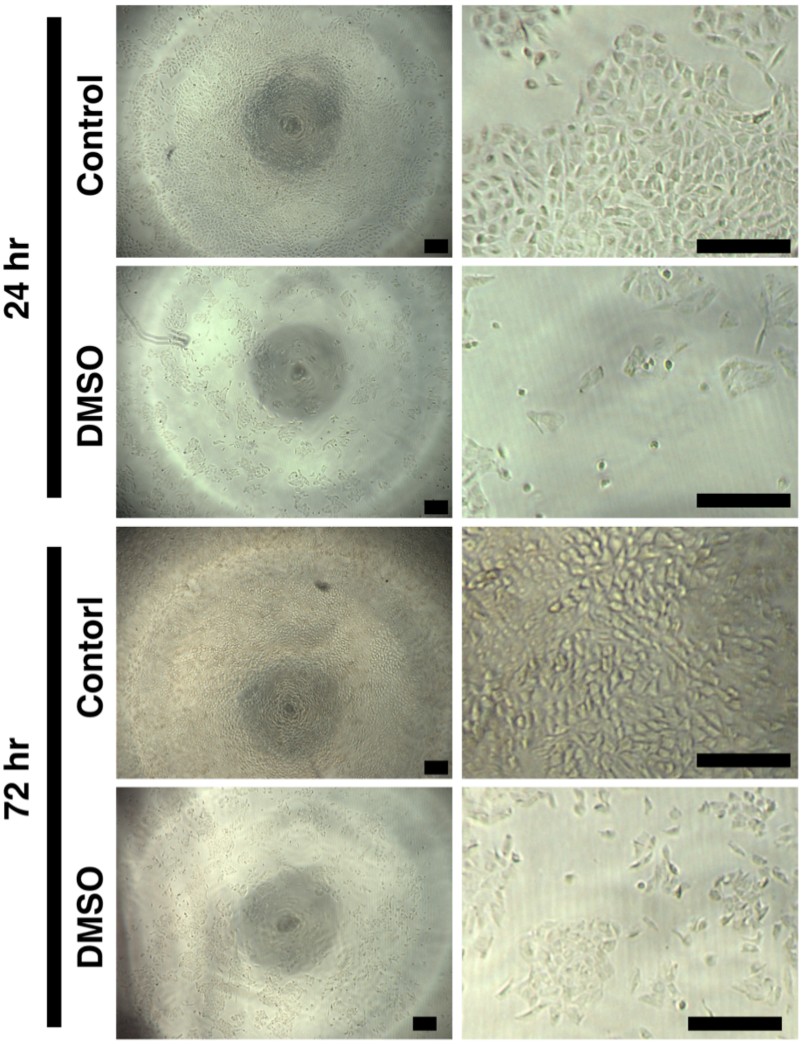

Figure S13. Optical microscopy images of control A549 cells and cells incubated DMSO. A549 cells were incubated for 24 and 74 hr in a 37 °C incubator supplied with 5 % CO_2. Scale bar = 5 μm. No concentrated black structures were found in control cells. DMSO was used as positive toxicity controls, as shown in the figures, most of the cells were died and detached from the plate when treated with DMSO.

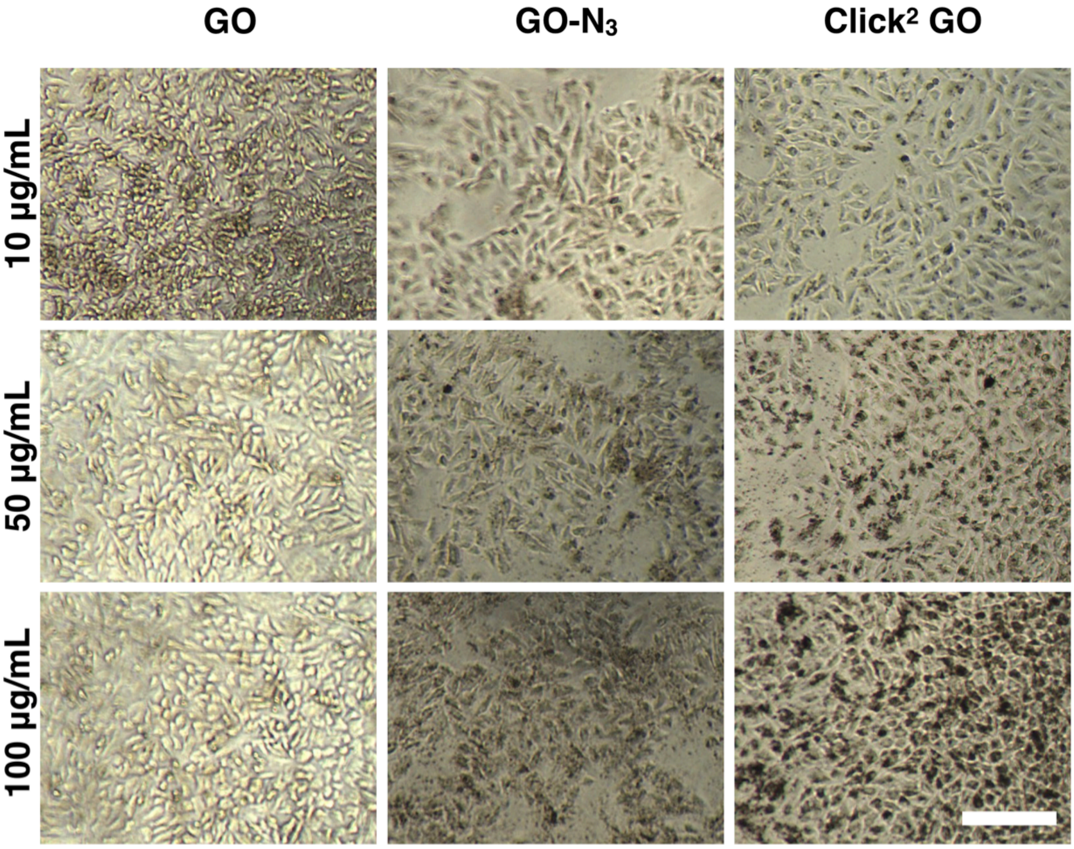

Figure S14. Optical microscopy images of A549 cells incubated with GO, GO-N_3 and Click^2 GO for 24 hr. GO, GO-N_3 and Click^2 GO were incubated with A549 cells at 10, 50, and 100 μg/mL for 24 hr in a 37 °C incubator supplied with 5 % CO_2. Scale bar = 5 μm. Graphene based materials seem to have minimum effect on cell growth. GO-N_3 and Click^2 GO showed a better cellular uptake profile when compared to plain GO sheets. Among the three, Click^2 GO appeared to internalise the cell very efficiently across the three concentrations at 24 hr time point, as reflected by the increasingly concentrated black pattern that matched the cell distribution. GO-N_3 also showed some degree of internalisation but less than the Click^2 GO.

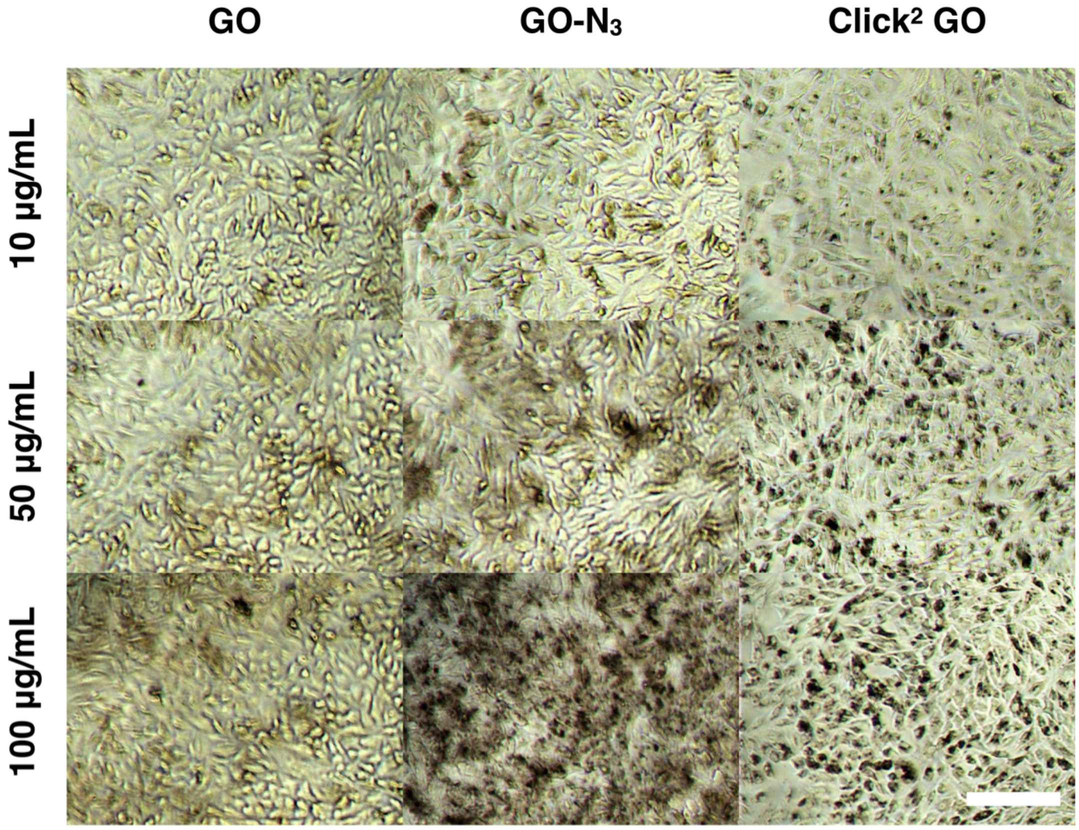

Figure S15. Optical microscopy images of A549 cells incubated with GO, GO-N_3 and Click[2] GO for 72 hr. GO, GO-N_3 and Click[2] GO were incubated with A549 cells at 10, 50, and 100 μg/mL for 72 hr in a 37 °C incubator supplied with 5 % CO_2. Scale bar = 5 μm.

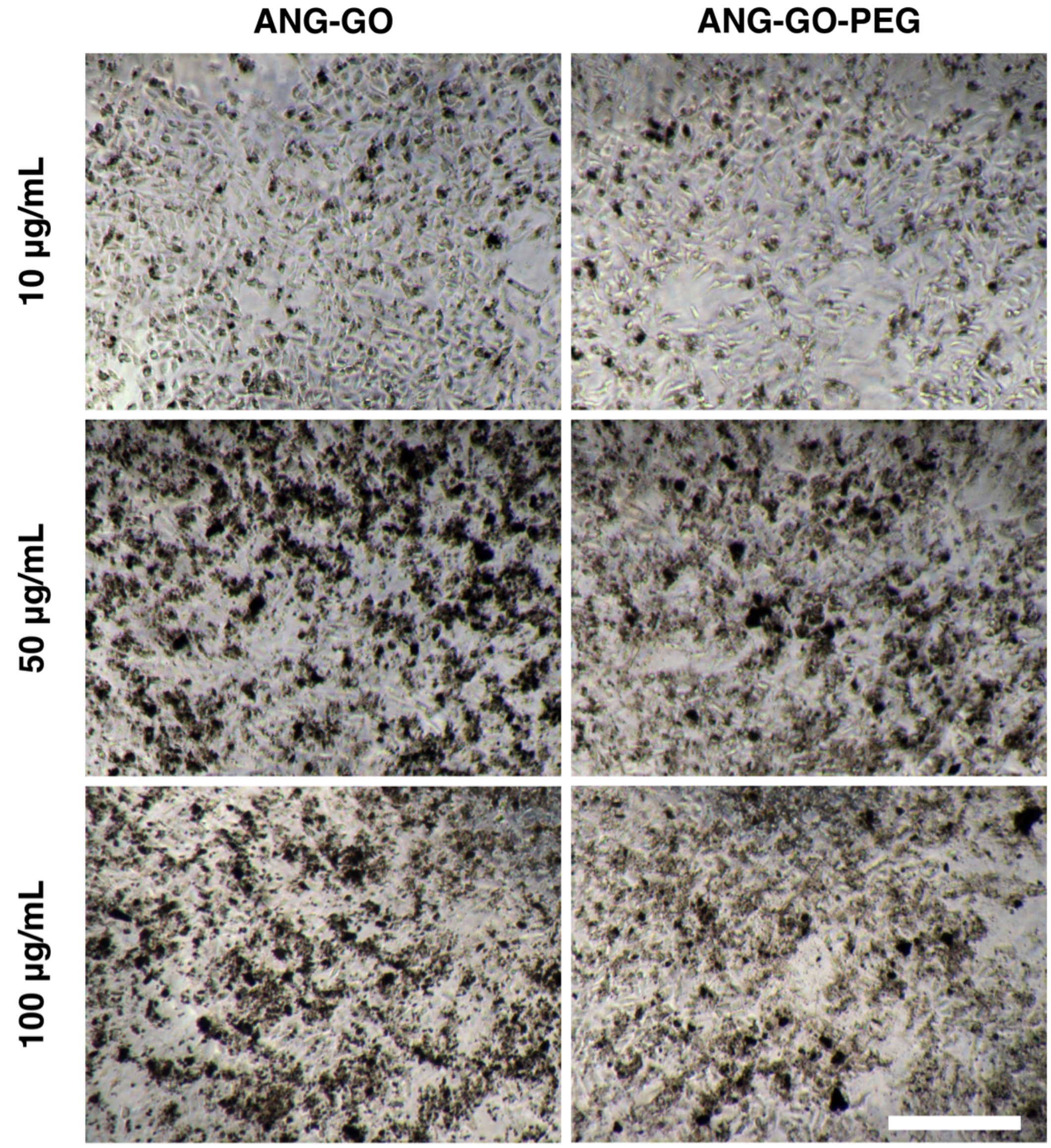

Figure S16. Optical microscopy images of A549 cells incubated with ANG-GO and ANG-GO-PEG for 24 hr. ANG-GO and ANG-GO-PEG were incubated with A549 cells at 10, 50, and 100 µg/mL for 24 hr in a 37 °C incubator supplied with 5 % CO_2. Scale bar = 5 µm.

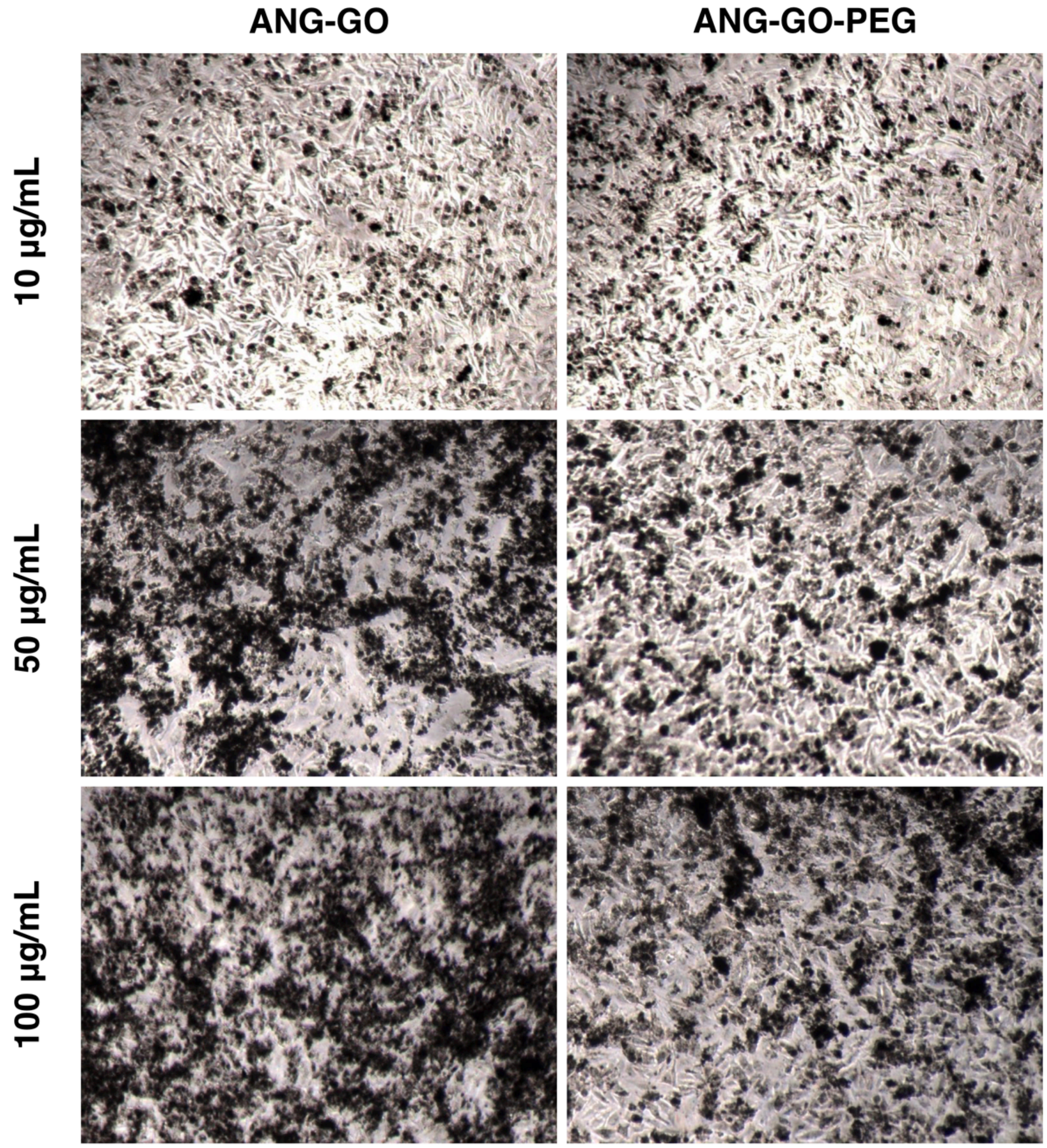

Figure S17. Optical microscopy images of A549 cells incubated with ANG-GO and ANG-GO-PEG for 72 hr. ANG-GO and ANG-GO-PEG were incubated with A549 cells at 10, 50, and 100 μg/mL for 72 hr in a 37 °C incubator supplied with 5 % CO_2. Scale bar = 5 μm.

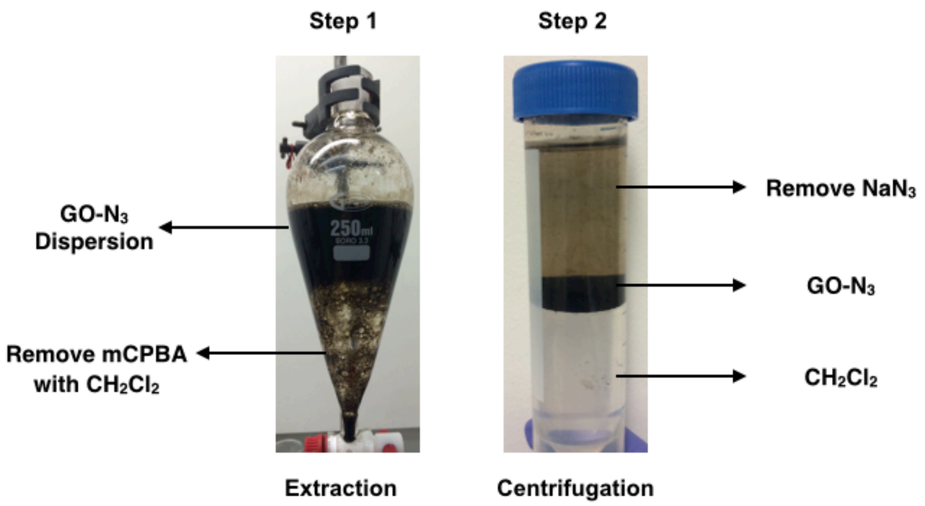

Figure S18: Purification of $GO\text{-}N_3$ by washing/centrifugation.

Table S1: Modified Hummer's Method v.s. modified Kovtyukhova-Hummer's method.

	Method 1[a]	Method 2[b]
Starting Graphite (mg)	4000	300
End product GO (mg)	5470	2
GO Yield[c]	55.63%	-

[a]Modified Kovtyukhova-Hummer's method.

[b]Modified Hummer's method.

[c]Calculated using **eq. S3**

Table S2: Elemental analysis of pre-oxidised graphite (po-G) and GO samples.

	Element (%)[a]		Atomic Mass	Element (w/w%)[b]	
	po-G	GO		po-G	GO
C	92.82	47.35	12	91.73	41.24
H	0.95	2.17	1	0.08	0.16
N	<0.10	<0.10	14	<0.10	<0.10
O[c]	6.13	50.38	16	8.08	58.50

[a]Measured by elemental analysis.

[b]Calculated using **eq. S2** (**SI** methods).

[c] O % was estimated by subtraction.

Table S3. Elemental analysis of GO-N_3 (± mCPBA).

Sample	Elements (%) [a]		
	C	H	N
GO (control)	47.35	2.17	<0.10
GO-N_3 (− mCPBA)	47.04	2.64	0.71
GO-N_3 (+ mCPBA)	45.62	2.50	0.81

[a] $n = 2$

Table S4: Thermogravimetric analysis of GO.

	Linear Ranges		Wt. Loss	Slope
	Temperature (°C)	Residual Weight (%)	(%)	ΔWt. (%) ∕ Δ°C
Ia	150 ~ 166	96 ~ 88	8	− 0.50
IIa	246 ~ 370	79 ~ 72	7	− 0.06
IIIa	370 ~ 604	72 ~ 63	9	− 0.04
IVa	790 ~ 978	52 ~ 35	17	− 0.09

Table S5: Thermogravimetric analysis of GO-N_3.

	Linear Ranges		Wt. Loss	Slope
	Temperature (°C)	Residual Weight (%)	(%)	ΔWt. (%) ⁄ Δ°C
Ib	182 ~ 208	91 ~ 77	15	− 0.57
IIb	231 ~ 410	73 ~ 64	9	− 0.05
IIIb	410 ~ 846	64 ~ 49	16	− 0.04
IVb	891 ~ 978	46 ~ 40	6	− 0.07

Table S6: Thermogravimetric analysis of Click^2 GO.

	Linear Ranges		Wt. Loss	Slope
	Temperature (°C)	Residual Weight (%)	(%)	ΔWt. (%) ⁄ Δ°C
Ic	198 ~ 227	90 ~ 73	17	− 0.59
IIc	336 ~ 978	66 ~ 46	20	− 0.03

References:

1 H. Ali-Boucetta, D. Bitounis, R. Raveendran-Nair, A. Servant, J. Van den Bossche and K. Kostarelos, *Adv. Healthc. Mater.*, 2013, **2**, 433–441.

2 N. I. Kovtyukhova, P. J. Ollivier, B. R. Martin, T. E. Mallouk, S. A. Chizhik, E. V. Buzaneva and A. D. Gorchinskiy, *Chem. Mater.*, 1999, **11**, 771–778.

3 W. Steglich and B. Neises, *Angew. Chem. Int. Ed.*, 1978, **17**, 522–524.

4 E. D. Goddard-Borger and R. V. Stick, *Org. Lett.*, 2007, **9**, 3797–3800.
